# Gut microbiome and metabolic activity in type 1 diabetes: An analysis based on the presence of GADA

**DOI:** 10.3389/fendo.2022.938358

**Published:** 2022-09-30

**Authors:** Sihui Luo, Tong Yue, Ziyu Liu, Daizhi Yang, Mengyun Xu, Yu Ding, Weiwei Jiang, Wen Xu, Jinhua Yan, Jianping Weng, Xueying Zheng

**Affiliations:** ^1^ Department of Endocrinology, the First Affiliated Hospital of USTC, Division of Life Sciences and Medicine, University of Science and Technology of China, Hefei, China; ^2^ Department of Endocrinology and Metabolism, The Third Affiliated Hospital of Sun Yat-sen University, Guangzhou, China

**Keywords:** T1D (type 1 diabetes), GADA, gut microbiome, serum metabolites, tryptophan metabolism

## Abstract

**Objective:**

Type 1 diabetes (T1D) progression is affected by circulating glutamic acid decarboxylase antibody (GADA) that persist for many years. This study aimed at investigating whether and how the gut microbiome and its correlated metabolites change in T1D with the presence of GADA.

**Methods:**

We used a radiobinding assay to measure GADA titers and identify the 49 T1D patients with GADA+ and 52 T1D patients with GADA-. The fresh feces and serum were analyzed using 16S rRNA gene sequencing and GC/MS. Then gut microbiome and serum metabolites were compared between the GADA+ patients and the GADA- patients. The association between gut microbial community and metabolites was assessed using the Spearman’s rank correlation.

**Results:**

The gut microbiome in diversity, composition, and function differed between these two groups. The abundance of genus *Alistipes*, *Ruminococcus* significantly increased in patients with GADA+ compared to that observed in the samples of GADA-. There were 54 significantly altered serum metabolites associated with tryptophan metabolism, phenylalanine, and tyrosine biosynthesis in individuals with GADA+ compared with those of GADA-For the serum metabolites, compared with those of GADA-, there were 54 significantly different metabolites with tryptophan metabolism, phenylalanine, and tyrosine and tryptophan biosynthesis decreased in individuals with GADA+. The abundance of *Alistipes* was positively correlated with altered metabolites involved in tryptophan metabolism.

**Conclusion:**

We demonstrate that T1D patients with GADA+ are characterised by aberrant profiles of gut microbiota and serum metabolites. The abundance of *Alistipes* disturbances may participate in the development of T1D patients with GADA by modulating the host’s tryptophan metabolism. These findings extend our insights into the association between the gut microbiota and tryptophan metabolism and GADA and might be targeted for preventing the development of T1D.

## Introduction

Type 1 diabetes mellitus (T1D) is an autoimmune disease characterized by pancreatic β-cells being destroyed (1). Autoimmunity is heterogeneous in the pathogenesis of T1D, and the presence of autoantibodies is considered a potential feature for classification (2, 3). Among the autoantibodies, glutamic acid decarboxylase antibody (GADA) is known for its early appearance, highest positivity rate, and most extended duration, and is often used in β-cell function prediction (4). During the follow-up of several longitudinal cohorts of T1D patients, GADA titers are shown to be associated with various persistent autoimmunity activity levels and disease development (5, 6). Patients who are GADA positive are more likely to have multiple autoimmune diseases, such as Hashimoto’s thyroiditis and gastritis (7, 8). However, the underlying biological mechanisms of T1D patients with different GADA phenotypes is not completely understood.

There has been an interest that the gut microbiota is an important environmental factor contributing to patients with autoimmune diseases, including rheumatoid arthritis (RA) and T1D (9, 10). Previous studies have shown that alpha-diversity decreased, accompanied by spikes in inflammation-favoring organisms and subsequent *E. coli* depletion prior to seroconversion was observed in patients with type 1 diabetes during disease progression (11, 12). Besides, the human gut microbiome composition and metabolic pathways may influence host metabolism and inflammation (13, 14). Given that the gut microbes have implications for host metabolic pathways, beta diversity plasma concentrations of indoxyl sulphate and L-citrulline differed in T1D without and with stratification by albuminuria (15).. The changes of these gut microbiota and the disturbance of serum metabolism provide a new understanding of the pathogenesis of T1D. Accumulating evidence indicates gut microbiota composition and function change might be involved in the progression from β-cell autoimmunity to clinical disease emerge after the appearance of autoantibodies (16, 17). However, the variation in how microbiota and serum metabolites may link to the metabolism of T1D with different immunotypes (without/with GADA) has not been investigated.

Here, we aimed to examine the gut microbiome composition and serum metabolites in individuals in different GADA subgroups. Furthermore, we looked into the interaction between GADA-associated microbiota and metabolism, and also their potential effect on the pathogenesis of T1D.

## Materials and methods

### Study population and clinical measures

All participants were recruited from the Third Affiliated Hospital of Sun Yat-sen University (Guangzhou, Guangdong, China). All patients with T1D were participants of the T1D China Registry Study (ChiCTR2000034642) (18). Inclusion criteria were patients who were diagnosed with T1D according to the previous report (18) and aged were >18 years (19). Exclusion criteria were: (1) use of antibiotics in the three months before study enrollment; (2) significant acute or other chronic illnesses including diseases of the digestive tract, severe psychiatric diseases; (3) abdominal surgery history; (4) dietary supplements including prebiotic, probiotic, or fiber-rich supplements within four weeks before the sample collection; and (5) dietary practices that are known to affect the gut microbiota, such as a weight-loss diet or a diet that is entirely composed of vegetables. Healthy controls were age- and sex-matched volunteers recruited from public advertisements.

GADA were detected from fasting serum with radio binding assay in duplicate following the standards of the Islet Autoantibody Standardization Program. The GADA positivity cutoff value was set at 0.05, which means that a test value over 0.05 was considered positive (20). We collected all participants’ information from the questionnaires and clinical examination, including but not limited to demographic and clinically relevant data. All diabetic patients were given structuralized diabetes education and were required to follow a diabetes diet. A food frequency questionnaire was collected to determine the dietary intake history. The study protocol conformed to the 1975 Declaration of Helsinki ethical guidelines, and all participating universities acquired Institutional Review Board approval, and the study received signed informed consent.

### DNA preparation and sequencing

Fresh feces and plasma samples were collected from participants in our hospital. The sample tubes were frozen and stored at −80°C immediately until further analysis. The microbial DNA was extracted from approximately 220 mg of feces using the QIAamp DNA Stool Mini Kit (Qiagen, Germany) following the manufacturer’s instructions. PCR using primers, 515F (5’-GTGCCAGCMGCCGCGGTAA-3’) and 806R (5’-GGACTACHVGGGTWTCTAAT-3’) were used to amplify the regions V4 of bacterial 16S rRNA gene. The PCR reactions were 50 μl reaction in triplicate. The PCR products were purified using Agencourt AMPure XP beads and eluted in an elution buffer. Purified amplicons were quantified using the Agilent Technologies 2100 bioanalyser and paired end sequenced (2 × 250) following the standard pipelines on an Illumina MiSeq platform (BGI, Shenzhen, China).

### 16s rRNA gene sequencing and bioinformatics analysis

Raw fastq files procession and analysis were conducted using the Quantitative Insights Into Microbial Ecology (QIIME2) platform (21). At each location with more than three consecutive bases, the 250 bp readings were clipped, resulting in an average quality score of 20. The Fast Length Adjustment of Short Reads (FLASH, v1.2.11) program was used to add paired-end reads to tags (22). Using UPARSE (v7.0.1090) with a cutoff value of 97%, the tags were grouped into OTUs, and chimera sequences were validated with UCHIME (v4.2.40) and excluded from further analysis. The remaining high-quality sequences were categorized into operational amplicon sequence variants (ASVs) at 100% similarity by DADA2 algorithm (23). The α-diversity was calculated at the gene abundance, including community richness indexes (Ace and Chao1) and community diversity indexes (Shannon and Simpson). The Bray-Curtis distance method was used to estimate β-diversity, which was reported using the principal coordinate analysis (PCoA); the difference between groups was examined using the permutational multivariate analysis of variance (PERMANOVA). On the normalized taxon composition, Linear discriminant analysis effect size (LEfSe) analysis was performed on a web server (http://huttenhower.sph.harvard.edu/galaxy) using linear discriminant analysis (LDA) score (24). KEGG functional predictions were generated using the PICRUSt algorithm (25).

### Serum metabolome analysis

The serum metabolite extracts were analyzed on LC-MS/MS technology. High-resolution mass spectrometer Q Exactive (Thermo Fisher Scientific, USA) collected positive and negative ions data. The raw LC-MS data were handled with The Compound Discoverer 3.1 (Thermo Fisher Scientific, USA) software, and multidimensional statistical analysis was performed PCA and OPLS-DA analyses. The variable importance projection (VIP) value, the fold change, and the Student’s test were used to screen for differential metabolites. The Enrichment analysis of the KEGG metabolic pathway based on the significantly altered metabolites was performed with MetaboAnalyst 5.0 (26). The three databases were searched to identify further and validate the differential metabolites, including Human Metabolome Database (HMDB), Kyoto Encyclopedia of Genes and Genomes (KEGG), and mzCloud.

### Statistical analysis

χ^2^ test for categorical variables, or a one-way analysis of variance (ANOVA) for continuous variables, were performed in comparisons among groups. And unpaired Student’s t-test was adopted in multiple comparisons. The Kruskal–Wallis test was performed in comparisons between groups for non-normally distributed variables. Data are presented as the mean ± SEM. The differential abundance of metabolites and genera was tested by Kruskal–Wallis test The network plot of metabolites and microbes was based on M^2^IA (27). The correlation between the metabolites and microbiota was estimated using Spearman’s rank correlation was performed in R version 4.0.2. *P*-value < 0.05 were considered statistically significant.

## Results

### Clinical characteristics in participants

We collected the serum samples from Chinese participants, including 101 T1D patients and 38 healthy controls. The clinical characteristics of the T1D patients are summarized in **Table 1**, and the detailed information including GADA titers among the three groups is shown in **Supplementary Table S1**. There were no significant differences in age and body mass index (BMI) between patients with GADA+ (age 27.10 ± 11.34 years; BMI, 20.44 ± 2.65 kg/m^2^), and patients with GADA- (age 26.62 ± 12.05 years; BMI, 20.35 ± 2.57 kg/m^2^). Moreover, there were no group differences in serum levels (mmol/L) of TC, TG, LDL-C, HDL-C, and glycemic control. However, patients with GADA+ had lower concentrations of fasting C-peptide than those in the patients with GADA- group (0.026 ± 0.29 vs 0.048 ± 0.06, pmol/L; *p* < 0.002).

**Table 1:** Characteristics of the participants.

	T1D		HCs (n=38)	*P-value*
	GADA- (n=52)	GADA+ (n=49)	
Sex (Women, %)	(29,55.77%)	(30,61.22%)	(24,63.16%)	0.578
Age, years	26.62 (12.05)	27.10 (11.34)	27.03 (11.30)	0.835
Diabetes Duration, years	10.21 (6.10)	11.42 (5.82)	─	0.237
BMI,kg/m2	20.35 (2.57)	20.44 (2.65)	21.58 (23.48)	0.852
HbA1c (%)	7.70 (2.04)	7.56 (1.48)	5.24 (0.31)	0.676
TC (mmol/L)	4.63 (0.86)	4.80 (0.82)	4.66 (0.80)	0.294
TG (mmol/L)	0.76 (0.33)	0.75 (0.32)	0.96 (0.50)	0.786
LDL-C (mmol/L)	2.69 (0.80)	2.85 (0.66)	2.78 (0.70)	0.183
HDL-C (mmol/L)	1.50 (0.25)	1.57 (0.32)	1.29 (0.24)	0.227
Fasting C-peptide (pmol/L)	0.048 (0.06)	0.026 (0.29)	─	0.002
Insulin regimen			─	1.000
MDI	25 (48.1)	23 (46.9)	─	
CSII	26 (50.0)	25 (51.0)	─	
BD	1 (1.92)	1 (2.04)	─	
Insulin dosage (u/kg)	0.75 (0.27)	0.73 (0.19)		0.665

Data are presented as mean (SD) or n (%).MDI: basal bolus, long-acting plus rapid insulin with meals; CSII: continuous subcutaneous insulin infusion; BD: twice-daily insulin (breakfast and evening meal). P-values were compared by the two groups (GADA-; GADA+). HCs, Healthy Controls; BMI, body mass index; TC, total cholesterol; TG, triglyceride; LDL-C, low-density lipoprotein cholesterol; HDL-C, high-density lipoprotein cholesterol; T test/ANOVA notes needed.

### The gut microbiome community profiling in T1D patients

**Figure 1:**
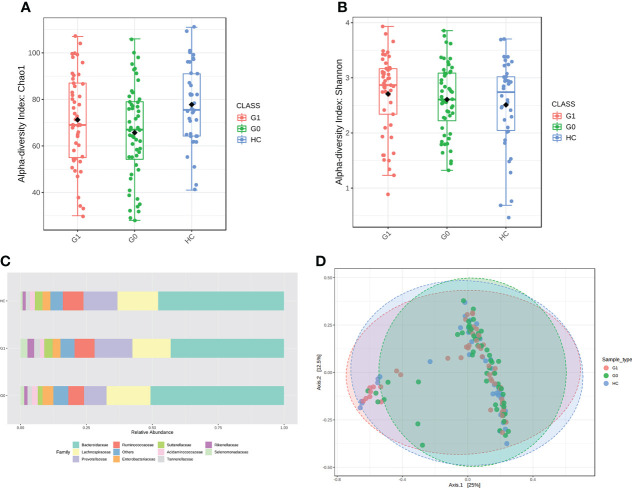
Results of diversity and structure profile. **(A, B)** Box plot of differences in α-diversity of the gut microbiota of Chao1 **(A)** and Shannon index **(B)** respectively, the difference of Chao1 index (ANOVA, p-value: 0.0148, F-value: 4.349), the difference of Shannon index (ANOVA, p-value: 0.439, F-value: 0.827) among the three study groups. **(C)** The stacked bar plots at the family level. **(D)** PCoA plot of β-diversity of the Bray–Curtis index shows microbial communities’ distance. (Permutational multivariate analysis of variance (PERMANOVA), F= 1.3449, R-squared= 0.019364; p< 0.128]. G0, GADA-; G1, GADA+.

We assessed the α-diversity and the β-diversity to investigate whether the gut microbial dysbiosis differed between HCs and T1D patients with GADA- or GADA+ in diversity and structure. HCs showed the highest level of microbial community diversity in contrast to the T1D patients, which was reflected in the Chao1 index (**Figure 1A**). However, the Shannon and Simpson indexes result did not reach the statistically significant threshold (**Figure 1B**, **Figure S1**). The Fisher indexes suggested that the gut microbiome community richness and species number differed between the GADA-, GADA+, and HCs groups (**Figure S1**). Next, we performed β-diversity based on the Bray-Curtis dissimilarity to explore the microbial composition among three groups (**Figure 1D**). No statistically significant differences were observed in the β-diversity of the gut microbiota among the GADA-, GADA+, and HCs groups.

We calculated all taxonomic abundance to analyze the abundance of similarity in bacterial taxonomy among the three groups. *Actinobacteriota*, *Bacteroidetes*, *Desulfobacterota*, *Firmicutes*, and *Proteobacteria* constituted most of the individuals’ gut microbiota at the phylum level (**Table S2**). As shown in **Figure 1C**, the top 10 abundant gut microbiota at the family level, which are different panels among the three groups. Twelve genera(*Erysipelotrichaceae_UCG_003*, *Coprococcus*, *Anaerostipes*, *Streptococcus Ruminococcus_torques*, *Blautia*, *Oscillibacter*, *Sutterella*, *Intestinibacter*, *Ruminococcus_gnavus*, *Veillonella*, and *Dorea*) were enriched in T1D patients compared with HC individuals, of which the majority pertained to the *Ruminococcu*s, and three genera (*Oscillospiraceae*, *Colidextribacter*, and *Butyricicoccus*) were depleted in individuals with type 1 diabetes. The relative abundance of *Alistipes*, *Ruminococcu*s was significantly enriched in the GADA+ group compared with the GADA- (*p* < 0.05), but the relative abundance of these genera did not differ between GADA- and HC individuals (**Figure S2**). These findings revealed gut microbial diversity and structure differences among T1D patients and HCs.

### Functional characterization of the gut microbiome in T1D patients with GADA

**Figure 2:**
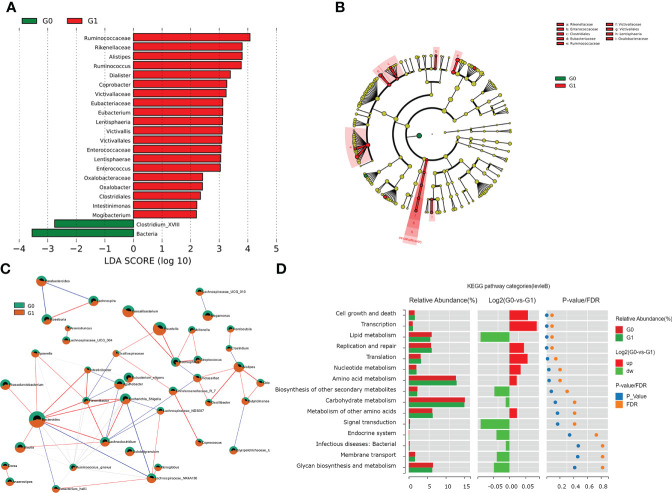
Results of gut microbial characteristics and correlations. **(A)** Histogram of LDA value distribution, list ranked by LDA score. **(B)**The cladogram shows different taxonomic levels between GADA+ or GADA- group. **(C)** Correlation among the differentially abundant microbiota in T1D patients. Nodes with correlations between circles represent positive (represented by red lines) and negative correlation (represented by blue lines). (Circle with red part: G1; circle with green part). **(D)** Wilcoxon test of pathway differences between GADA+ and GADA-groups. The histogram shows the relative abundance for pathway in each group. The middle panel is a histogram of the log2 value of the mean relative abundance ratio in the two groups of the same pathway. The P and FDR values obtained by the Wilcoxon test are shown on the right panel.

Among the individuals with type 1 diabetes, we performed LEfSe analysis to determine different microbial compositions at all taxonomic levels between the two groups. In patients with GADA+, there were 22 bacterial taxonomic clades (20 increased and 2 decreased) detected by LDA score showing statistically significant differences, compared with those in GADA- patients (**Figure 2A**, **Table S3**). Compared with patients with GADA-, patients with GADA+ showed higher abundances of *Ruminococcus*, *Alistipes*, *Dialister*, *Coprobacter*, *Eubacterium*, *Victivallis*, *Enterococcus*, *Oxalobacter*, *Intestinimonas*, and *Mogibacterium* and lower abundances of the *Clostridium_XVIII* at the genus level (**Figure 2A**, **Table S3**). The abundant bacteria at the family level in GADA+ patients included Rikenellaceae, a family of Bacteroidetes; and *Ruminococcaceae*, *Eubacteriaceae*, and *Enterococcaceae*, belonging to Firmicutes (**Figure 2B**). We used the SparCC algorithm to explore the relationships between dominating microbiota at the ecological dimension. In an altered genus-genus co-occurrence network, *Bacteroides*, *Blautia*, *Phascolarctobacterium*, *Ruminococcus*, and *Lachnoclostridium* exhibited positive interactions in T1D patients (**Figure 2C**). In the T1D patients with GADA+, we observed that *Alispties* were key genera that play essential roles in the composition and function of the intestinal micro-environment.

We conducted the KEGG pathway analysis using the PICRUSt2 algorithm to explore the link between the dominating microbiota and the functional profile of a microbial community. Compared with patients with GADA-, the abundance of pathways linked with cell growth and death, transcription, and nucleotide metabolism were significantly increased in patients with GADA+ (**Figure 2D**, **Table S4**). Further analysis found that the microbial gene function related to bile acid biosynthesis, and phenylalanine metabolism was decreased in patients with GADA+ (**Figure S3**, **Table S4**).

### OPLS−DA and metabolites identification in serum from T1D patients

**Figure 3:**
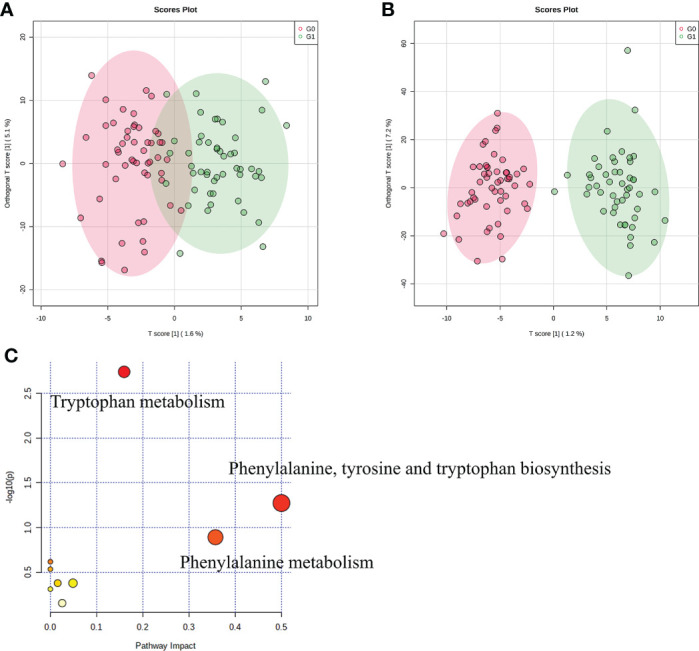
Profiles of serum metabolites. **(A, B)** The OPLS−DA score plots of serum samples from T1D patients in **(A)** negative ion mode and **(B)** positive ion mode. **(C)** KEGG pathway enrichment analysis of significantly altered metabolites. The size and color of each circle were obtained by pathway impact value and p-value, respectively.

We used LC-MS to compare the metabolic signatures of patients with GADA+ or GADA- to investigate how the gut microbiota modulates host metabolic pathways. The metabolites profile indicating global changes to serum metabolite composition in patients with GADA+ were significantly different from patients with GADA- (**Figures 3A, B**). The relative concentration of 54 significantly different serum metabolites (Fold change > 1.2, Fold change < 0.83, and p < 0.05) were identified by using the three databases (**Table S5**). 7 up-regulated and 47 down-regulated metabolites differed significantly in concentration between patients with GADA+ and GADA-.

Three metabolites related to tryptophan metabolism, including 3-hydroxyanthranilic acid, L-phenylalanine, and L-kynurenine, were lower in patients with GADA+ than those with GADA-. The KEGG pathway analysis showed that the altered serum metabolites were linked with tryptophan metabolism, phenylalanine, tyrosine and tryptophan biosynthesis and phenylalanine metabolism (**Figure 3C**). Most of the metabolites involved in tryptophan metabolism also be involved in phenylalanine, tyrosine, and tryptophan biosynthesis. For example, L-phenylalanine is a key node between the two metabolic pathways, broadly associated with tryptophan metabolism. Notably, we noticed a reduction of two metabolites involved in the pathway the reduced levels of tryptophan metabolism, namely L-phenylalanine and 5-hydroxyindoleacetate, in the T1D patients with GADA+. (**Figure S4**).

### Co-occurrence network analysis of altered gut microbiota and serum metabolites

**Figure 4:**
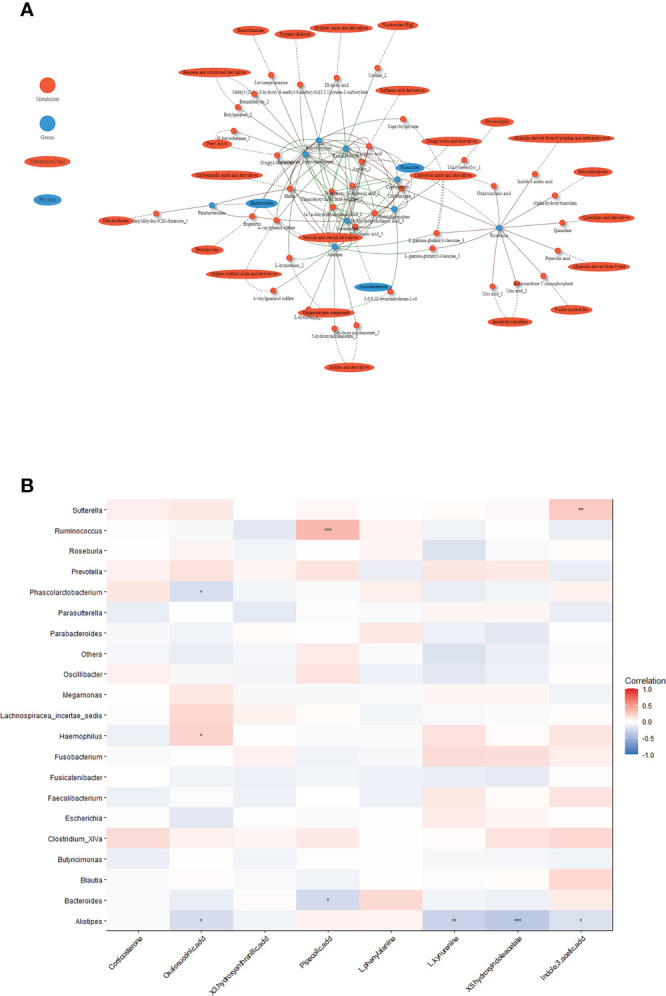
Associations between serum metabolites and altered gut microbiota. **(A)** Network of the Spearman’s rank correlation coefficient between metabolites class and gut microbes. (red circles: metabolites; blue ovals: microbes; red lines: positive correlation; green lines: negative correlation). **(B)** Heatmap of correlation between altered metabolites and gut bacteria at the genus level. The colors in the heatmap represent the positive (represented by pink) or negative correlation (represented by blue). The Benjamini-Hochberg false discovery rate was used to correct p values for multiple testing. *p<0.05, **p<0.01, ***p<0.001.

We constructed a co-occurrence network to explore potential associations between altered gut microbiota and serum metabolites. We found that *Alistipes*, *Ruminococcus*, *Butyricimonas* formed strong co-occurring relationships with steroids and steroids derivatives (3a,7a-dihydroxycholanoic acid, 3b-hydroxy-5-cholenoic acid), fatty acyls (3-(acetylsulfanyl)-2-(sulfanylmethyl) propanoic acid), and sulfamic acid derivatives (cyclamic acid) (**Figure 4A**). Additionally, we used the top changed metabolites for spearman correlation analysis with genus abundance. As shown in **Figure 4B**, the change in *Ruminococcus* was positively associated with the change in pipecolic acid (r=0.34, p<0.001), whereas the change in *Alistipes* was negatively associated with the changes in 5-hydroxyindoleacetate (r=-0.35, p<0.001), which was involved in tryptophan metabolism (**Figure 4B**, **Table S4**).

## Discussion

In the present study, we included 49 T1D patients with GADA+ and 52 T1D patients with GADA-. We examined gut microbiome composition and serum metabolites to explore the potential association of microbiota and metabolite in T1D patient with different GADA levels. Among individuals with type 1 diabetes, we found that the gut microbiome and serum metabolites profile of GADA+ is distinct from GADA-. The abundance of *Alistipes* was negatively associated with serum metabolites involved in tryptophan metabolism. Integrated analysis of the microbiome-metabolome shows the correlation between microbiome and metabolites in T1D patients, driving or perpetuating the progression.

Small studies in humans have found the c-peptide levels drop after a diagnosis of (T1D), indicating that β cell function is deteriorating (28). In our study, focusing on the β cell function of T1D patients with different levels of GADA, we found that GADA+ patients have lower C-peptide concentrations than GADA- patients.

The gut microbiota may increase gut permeability and regulate metabolism through disturbing immune system to impact the pathogenesis of T1D (3, 29). Previous studies have reported that T1D patients had significant disturbances in *Bacteroidetes/Firmicutes*, *Lactobacillus*, *Blautia*, and *Prevotella*, relative to healthy controls (17, 30–32). We found that 22 bacterial taxonomic clades (20 increased and 2 decreased) of gut bacteria differed significantly between individuals with type 1 diabetes stratified by levels of GADA. We found that the lower abundance of *Clostridium_XVIII* in GADA+ patients. *Clostridium* represent intestinal commensal bacteria, ferment a variety of benefit metabolites, especially butyrate, possess anti-inflammation effects, and maintain the intestinal immune system (33).

Differences in the first appearing autoantibody related to the average longitudinal abundance of *Ruminococcus* may be characterized by distinct microbial configurations (16). Moreover, GADA explains variation in the gut microbiota and strongly correlates with microbiota composition and structure (31). Our study found that patients with GADA+ showed a significant increase in the abundance of *Alistipes*, *Ruminococcus*. Inflammation correlated with an increased abundance of *Alistipes* and *Ruminococcus*(34). These interconnections might play vital roles in shaping the gut microbiome in T1D patients with GADA+. The studies of genus *Alistipes* that have provided conflicting findings have been protective effects for liver fibrosis, and cardiovascular disease *via* product short fatty acids (SCFA), whereas as potential drivers of intestinal barrier dysfunction and inflammation affect inflammatory bowel disease (IBD) (35). Some genus abundance and gene function changes may differ from previous studies because the microbiome is influenced by various factors, such as ethnicity, age, geography, and cultural differences. Together, these results suggest gut microbial dysbiosis may affect the host immune progression in T1D patients with GADA+.

We found that GADA+ patients were characterized by disturbances of gut microbiota and serum metabolites. Among the 54 differentially expressed serum metabolites, the reduced levels of 3-hydroxyanthranilic acid, L-phenylalanine, and L-kynurenine belong to tryptophan-related metabolites in T1D patients with GADA+. Tryptophan metabolism has become a vital part of cellular and organismal communication strategies, regulating immunity and intestinal homeostasis (36). It was reported that autoimmune diseases including IBD, Crohn’s disease (CD), and T1D were associated with a lower ratio of tryptophan metabolites (37–39).

An aberrant gut microbiota in individuals with GADA may contribute to lower tryptophan-related metabolites, which promotes progression of the disease. The tryptophan metabolites binding to the aryl hydrocarbon receptor (AHR), which promotes IL-22 secretion, improves the intestinal epithelial barrier, stimulates gastrointestinal motility, has anti-inflammatory properties, and may modulate gut microbial composition (40–42). Indole-3-acetic-acid, as a bacterial tryptophan metabolite, regulates intestinal immune function in combination with activation of AHR. In our study we found *Alistipes* and *Clostridium XlVa* was positively correlated with 5-hydroxyindoleacetate and Indole-3-acetic-acid (r=0.19, p=0.06). It is possible that this depletion of tryptophan metabolism in T1D patients with GADA+ by a T1D-associated dysbiosis microbiota (40). These findings suggest the possible association between gut microbial dysbiosis and tryptophan metabolism, which may provide another dimension to understanding the immune progression of T1D.

This study has some limitations. First, our study design was a cross-sectional analysis; we can only identify associations. It would also be interesting to systematically study the progression of disease dynamics in T1D patients by longitudinal multi-omics. In addition, we used 16S rDNA amplicon sequencing; a deep shotgun sequencing can give more insights into the function and specific species of gut microbiomes than 16S rDNA amplicon sequencing. We cannot conclusively determine whether the gut microbiome and metabolites dysbiosis is the etiology in T1D, which fecal transplantation experiments should further confirm. We expect future model experiments and longitudinal cohort studies of an altered gut microbiome and tryptophan metabolites in T1D to move towards mechanistic studies.

In conclusion, we provided the gut microbiome and serum metabolites profiles in T1D patients without and with GADA. In light of our results, we used multi-omics data and characterized the network of interactions between the microbes and serum metabolites that may play a critical role in the development of T1D. We found that gut microbiome disturbances may modulate the host’s tryptophan metabolism in T1D patients with GADA. We expect our exploratory study to spark longitudinal cohorts to confirm and expand intervention in human T1D heterogeneity.

## Data availability statement

The datasets presented in this study can be found in online repositories. The names of the repository/repositories and accession number(s) can be found below: https://www.ncbi.nlm.nih.gov/, PRJNA766410.

## Author contributions

Conception and design of the study: XZ, TY, SL, and JW. Acquisition of data was performed by XZ, TY, and SL. Collection of data: ZL, JY, DY, WX, WJ. Analysis and interpretation of data: TY, SL, MX, YD. All authors contributed to the article and approved the submitted version.

## Funding

This study was supported by grants from the National Natural Science Foundation of China (Grant No. 82100822), the Anhui Provincial Natural Science Foundation (2008085MH248, 2008085MH278), the Program for Innovative Research Team of The First Affiliated Hospital of USTC (CXGG02), and the Fundamental Research Funds for the Central Universities.

## Acknowledgments

We appreciate all the participants, on-site staff for participating in this research, and all research members of the Weng laboratory for scientific discussions concerning the manuscript.

## Conflict of interest

The authors declare that the research was conducted in the absence of any commercial or financial relationships that could be construed as a potential conflict of interest.

## Publisher’s note

All claims expressed in this article are solely those of the authors and do not necessarily represent those of their affiliated organizations, or those of the publisher, the editors and the reviewers. Any product that may be evaluated in this article, or claim that may be made by its manufacturer, is not guaranteed or endorsed by the publisher.

## References

[1] Katsarou A Gudbjörnsdottir S Rawshani A Dabelea D Bonifacio E Anderson BJ. Type 1 diabetes mellitus. Nat Rev Dis Primers (2017) 3:17016. doi: 10.1038/nrdp.2017.16 28358037

[2] Battaglia M Ahmed S Anderson MS Atkinson MA Becker D Bingley PJ. Introducing the endotype concept to address the challenge of disease heterogeneity in type 1 diabetes. Diabetes Care (2020) 43(1):5–12. doi: 10.2337/dc19-0880 31753960 PMC6925574

[3] Petrelli A Atkinson MA Pietropaolo M Giannoukakis N. Modulation of leukocytes of the innate arm of the immune system as a potential approach to prevent the onset and progression of type 1 diabetes. Diabetes (2021) 70(2):313–22. doi: 10.2337/dbi20-0026 PMC7881863 33472941

[4] Krischer JP Lynch KF Lernmark Å Hagopian WA Rewers MJ She JX. Genetic and environmental interactions modify the risk of diabetes-related autoimmunity by 6 years of age: The TEDDY study. Diabetes Care (2017) 40(9):1194–202. doi: 10.2337/dc17-0238 PMC5566280 28646072

[5] Sosenko JM Skyler JS Palmer JP Krischer JP Yu L Mahon J. The prediction of type 1 diabetes by multiple autoantibody levels and their incorporation into an autoantibody risk score in relatives of type 1 diabetic patients. Diabetes Care (2013) 36(9):2615–20. doi: 10.2337/dc13-0425 PMC3747899 23818528

[6] Liu L Li X Xiang Y Huang G Lin J Yang L. Latent autoimmune diabetes in adults with low-titer GAD antibodies: similar disease progression with type 2 diabetes: A nationwide, multicenter prospective study (LADA China study 3). Diabetes Care (2015) 38(1):16–21. doi: 10.2337/dc14-1770 25336751

[7] Gougourelas D Tsentidis C Koufadaki AM Koutsovasilis A Gougourela E Karanasios S. Associated autoimmunity in type 1 diabetes and latent autoimmune diabetes of adults: The role of glutamic-acid decarboxylase autoantibodies. Diabetes Res Clin Pract (2021) 175:108847. doi: 10.1016/j.diabres.2021.108847 33945840

[8] Kordonouri O Charpentier N Hartmann R. GADA positivity at onset of type 1 diabetes is a risk factor for the development of autoimmune thyroiditis. Pediatr Diabetes (2011) 12(1):31–3. doi: 10.1111/j.1399-5448.2010.00666.x 20723098

[9] Lee JY Mannaa M Kim Y Kim J Kim GT Seo YS. Comparative analysis of fecal microbiota composition between rheumatoid arthritis and osteoarthritis patients. Genes (Basel) (2019) 10(10):748. doi: 10.3390/genes10100748 PMC6827100 31557878

[10] Zhou H Sun L Zhang S Zhao X Gang X Wang G. Evaluating the causal role of gut microbiota in type 1 diabetes and its possible pathogenic mechanisms. Front Endocrinol (Lausanne) (2020) 11:125. doi: 10.3389/fendo.2020.00125 32265832 PMC7105744

[11] Tetz G Brown SM Hao Y Tetz V. Type 1 diabetes: an association between autoimmunity, the dynamics of gut amyloid-producing e. coli Their Phages Sci Rep (2019) 9(1):9685. doi: 10.1038/s41598-019-46087-x 31273267 PMC6609616

[12] Kostic AD Gevers D Siljander H Vatanen T Hyötyläinen T Hämäläinen AM. The dynamics of the human infant gut microbiome in development and in progression toward type 1 diabetes. Cell Host Microbe (2015) 17(2):260–73. doi: 10.1016/j.chom.2015.01.001 PMC4689191 25662751

[13] Wahlström A Sayin SI Marschall HU Bäckhed F. Intestinal crosstalk between bile acids and microbiota and its impact on host metabolism. Cell Metab (2016) 24(1):41–50. doi: 10.1016/j.cmet.2016.05.005 27320064

[14] Oh TG Kim SM Caussy C Fu T Guo J Bassirian S. A universal gut-Microbiome-Derived signature predicts cirrhosis. Cell Metab (2020) 32(5):878–888.e876. doi: 10.1016/j.cmet.2020.06.005 32610095 PMC7822714

[15] Winther SA Henriksen P Vogt JK Hansen TH Ahonen L Suvitaival T. Gut microbiota profile and selected plasma metabolites in type 1 diabetes without and with stratification by albuminuria. Diabetologia (2020) 63(12):2713–24. doi: 10.1007/s00125-020-05260-y 32886190

[16] Vatanen T Franzosa EA Schwager R Tripathi S Arthur TD Vehik K. The human gut microbiome in early-onset type 1 diabetes from the TEDDY study. Nature (2018) 562(7728):589–94. doi: 10.1038/s41586-018-0620-2 PMC6296767 30356183

[17] Huang Y Li SC Hu J Ruan HB Guo HM Zhang HH. Gut microbiota profiling in han Chinese with type 1 diabetes. Diabetes Res Clin Pract (2018) 141:256–63. doi: 10.1016/j.diabres.2018.04.032 29733871

[18] Weng J Zhou Z Guo L Zhu D Ji L Luo X. Incidence of type 1 diabetes in China, 2010-13: population based study. Bmj (2018) 360:j5295. doi: 10.1136/bmj.j5295 29298776 PMC5750780

[19] Yang D Deng H Luo G Wu G Lin S Yuan L. Demographic and clinical characteristics of patients with type 1 diabetes mellitus: A multicenter registry study in guangdong, China. J Diabetes (2016) 8(6):847–53. doi: 10.1111/1753-0407.12366 26663759

[20] Cheng J Yin M Tang X Yan X Xie Y He B. Residual β-cell function after 10 years of autoimmune type 1 diabetes: prevalence, possible determinants, and implications for metabolism. Ann Transl Med (2021) 9(8):650. doi: 10.21037/atm-20-7471 33987348 PMC8106063

[21] Bolyen E Rideout JR Dillon MR Bokulich NA Abnet CC Al-Ghalith GA. Reproducible, interactive, scalable and extensible microbiome data science using QIIME 2. Nat Biotechnol (2019) 37(8):852–7. doi: 10.1038/s41587-019-0209-9 PMC7015180 31341288

[22] Magoč T Salzberg SL. FLASH: fast length adjustment of short reads to improve genome assemblies. Bioinformatics (2011) 27(21):2957–63. doi: 10.1093/bioinformatics/btr507 PMC3198573 21903629

[23] Callahan BJ McMurdie PJ Rosen MJ Han AW Johnson AJ Holmes SP. DADA2: High-resolution sample inference from illumina amplicon data. Nat Methods (2016) 13(7):581–3. doi: 10.1038/nmeth.3869 PMC4927377 27214047

[24] Segata N Izard J Waldron L Gevers D Miropolsky L Garrett WS. Metagenomic biomarker discovery and explanation. Genome Biol (2011) 12(6):R60. doi: 10.1186/gb-2011-12-6-r60 21702898 PMC3218848

[25] Douglas GM Beiko RG Langille MGI. Predicting the functional potential of the microbiome from marker genes using PICRUSt. Methods Mol Biol (2018) 1849:169–77. doi: 10.1007/978-1-4939-8728-3_11 30298254

[26] Pang Z Chong J Zhou G de Lima Morais DA Chang L Barrette M. MetaboAnalyst 5.0: narrowing the gap between raw spectra and functional insights. Nucleic Acids Res (2021) 49(W1):W388–w396. doi: 10.1093/nar/gkab382 34019663 PMC8265181

[27] Ni Y Yu G Chen H Deng Y Wells PM Steves CJ. M2IA: a web server for microbiome and metabolome integrative analysis. Bioinformatics (2020) 36(11):3493–8. doi: 10.1093/bioinformatics/btaa188 32176258

[28] Lam A Dayan C Herold KC. A little help from residual β cells has long-lasting clinical benefits. J Clin Invest (2021) 131(3):e143683. doi: 10.1172/jci143683 PMC7843219 33529163

[29] Bosi E Molteni L Radaelli MG Folini L Fermo I Bazzigaluppi E. Increased intestinal permeability precedes clinical onset of type 1 diabetes. Diabetologia (2006) 49(12):2824–7. doi: 10.1007/s00125-006-0465-3 17028899

[30] Murri M Leiva I Gomez-Zumaquero JM Tinahones FJ Cardona F Soriguer F. Gut microbiota in children with type 1 diabetes differs from that in healthy children: a case-control study. BMC Med (2013) 11:46. doi: 10.1186/1741-7015-11-46 23433344 PMC3621820

[31] Leiva-Gea I Sánchez-Alcoholado L Martín-Tejedor B Castellano-Castillo D Moreno-Indias I Urda-Cardona A. Gut microbiota differs in composition and functionality between children with type 1 diabetes and MODY2 and healthy control subjects: A case-control study. Diabetes Care (2018) 41(11):2385–95. doi: 10.2337/dc18-0253 30224347

[32] Qi CJ Zhang Q Yu M Xu JP Zheng J Wang T. Imbalance of fecal microbiota at newly diagnosed type 1 diabetes in Chinese children. Chin Med J (Engl) (2016) 129(11):1298–304. doi: 10.4103/0366-6999.182841 PMC4894039 27231166

[33] Guo P Zhang K Ma X He P. Clostridium species as probiotics: potentials and challenges. J Anim Sci Biotechnol (2020) 11:24. doi: 10.1186/s40104-019-0402-1 32099648 PMC7031906

[34] Saulnier DM Riehle K Mistretta TA Diaz MA Mandal D Raza S. Gastrointestinal microbiome signatures of pediatric patients with irritable bowel syndrome. Gastroenterology (2011) 141(5):1782–91. doi: 10.1053/j.gastro.2011.06.072 PMC3417828 21741921

[35] Parker BJ Wearsch PA Veloo ACM Rodriguez-Palacios A. The genus alistipes: Gut bacteria with emerging implications to inflammation, cancer, and mental health. Front Immunol (2020) 11:906. doi: 10.3389/fimmu.2020.00906 32582143 PMC7296073

[36] Platten M Nollen EAA Röhrig UF Fallarino F Opitz CA. Tryptophan metabolism as a common therapeutic target in cancer, neurodegeneration and beyond. Nat Rev Drug Discovery (2019) 18(5):379–401. doi: 10.1038/s41573-019-0016-5 30760888

[37] Gürcü S Girgin G Yorulmaz G Kılıçarslan B Efe B Baydar T. Neopterin and biopterin levels and tryptophan degradation in patients with diabetes. Sci Rep (2020) 10(1):17025. doi: 10.1038/s41598-020-74183-w 33046801 PMC7552423

[38] Gupta NK Thaker AI Kanuri N Riehl TE Rowley CW Stenson WF. Serum analysis of tryptophan catabolism pathway: correlation with crohn's disease activity. Inflammation Bowel Dis (2012) 18(7):1214–20. doi: 10.1002/ibd.21849 PMC3235239 21823214

[39] Wlodarska M Luo C Kolde R d'Hennezel E Annand JW Heim CE. Indoleacrylic acid produced by commensal peptostreptococcus species suppresses inflammation. Cell Host Microbe (2017) 22(1):25–37.e26. doi: 10.1016/j.chom.2017.06.007 28704649 PMC5672633

[40] Roager HM Licht TR. Microbial tryptophan catabolites in health and disease. Nat Commun (2018) 9(1):3294. doi: 10.1038/s41467-018-05470-4 30120222 PMC6098093

[41] Dong F Hao F Murray IA Smith PB Koo I Tindall AM. Intestinal microbiota-derived tryptophan metabolites are predictive of ah receptor activity. Gut Microbes (2020) 12(1):1–24. doi: 10.1080/19490976.2020.1788899 PMC7524359 32783770

[42] Monteleone I Rizzo A Sarra M Sica G Sileri P Biancone L. Aryl hydrocarbon receptor-induced signals up-regulate IL-22 production and inhibit inflammation in the gastrointestinal tract. Gastroenterol 141(1) (2011) 237-248:248.e231. doi: 10.1053/j.gastro.2011.04.007 21600206

