# Supplementary material for: Gut microbiome and metabolic activity in type 1 diabetes: An analysis based on the presence of GADA
Source: Front Endocrinol (Lausanne). 2022 Sep 30;13:938358. doi: 10.3389/fendo.2022.938358 (PMC9563112; doi:10.3389/fendo.2022.938358)
Supplement: Supplementary file 1 [file DataSheet_1.docx]

Supplementary Material

## 1 Supplementary Figures


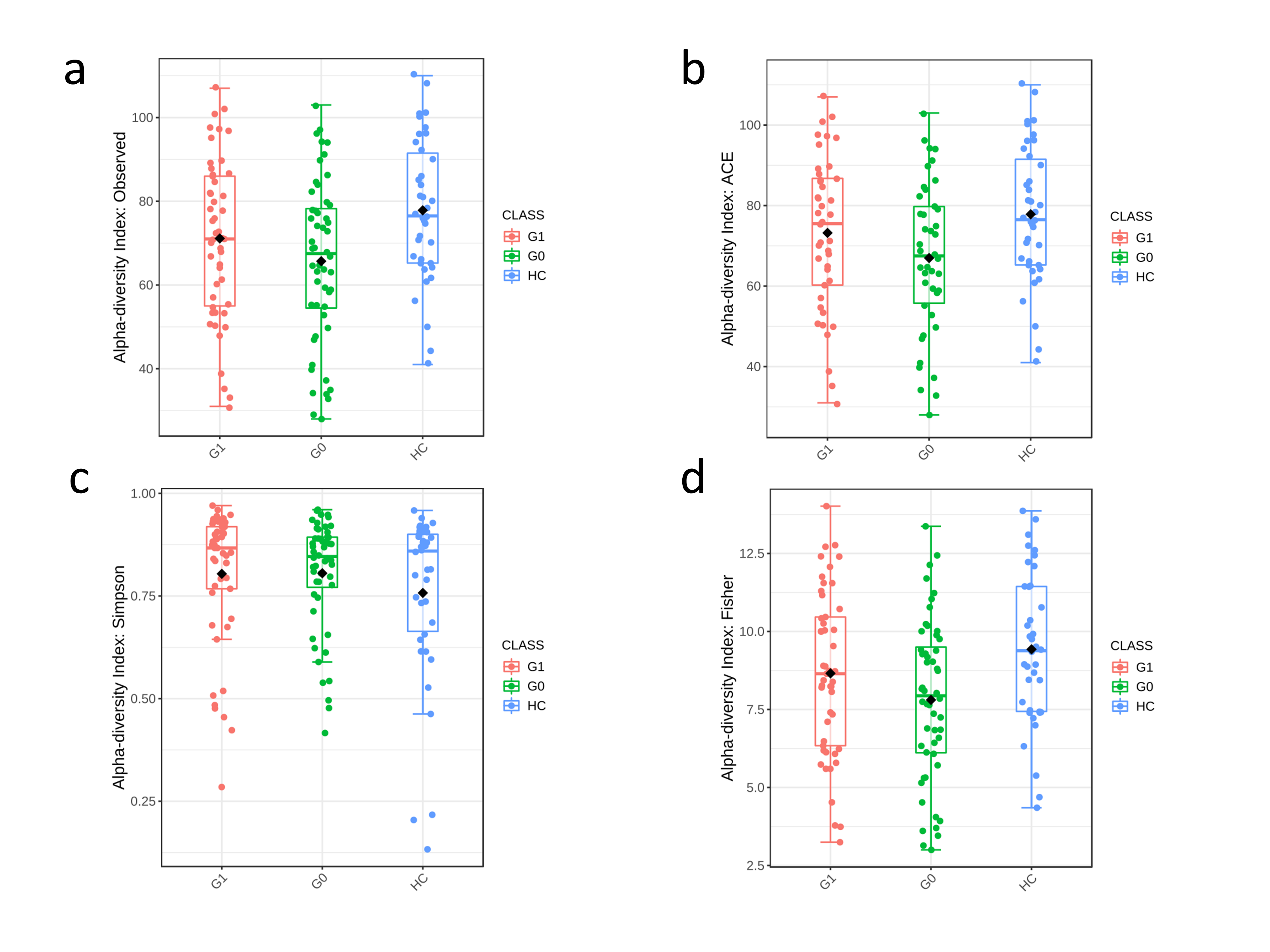


**Supplementary Figure S1. The boxplots of α-diversity of Obeserved (a), ACE (b), Simpson (c), and Fisher index (d).** The difference of observed index (ANOVA, p-value: 0.0148, F-value: 4.349), and Fisher index (ANOVA, p-value: 0.0141, F-value: 4.398) were significant between the three groups, but the difference of Ace index (ANOVA, p-value: 0.0526, F-value: 3.02) Simpson index were insignificant (ANOVA, p-value: 0.367, F-value: 1.01).


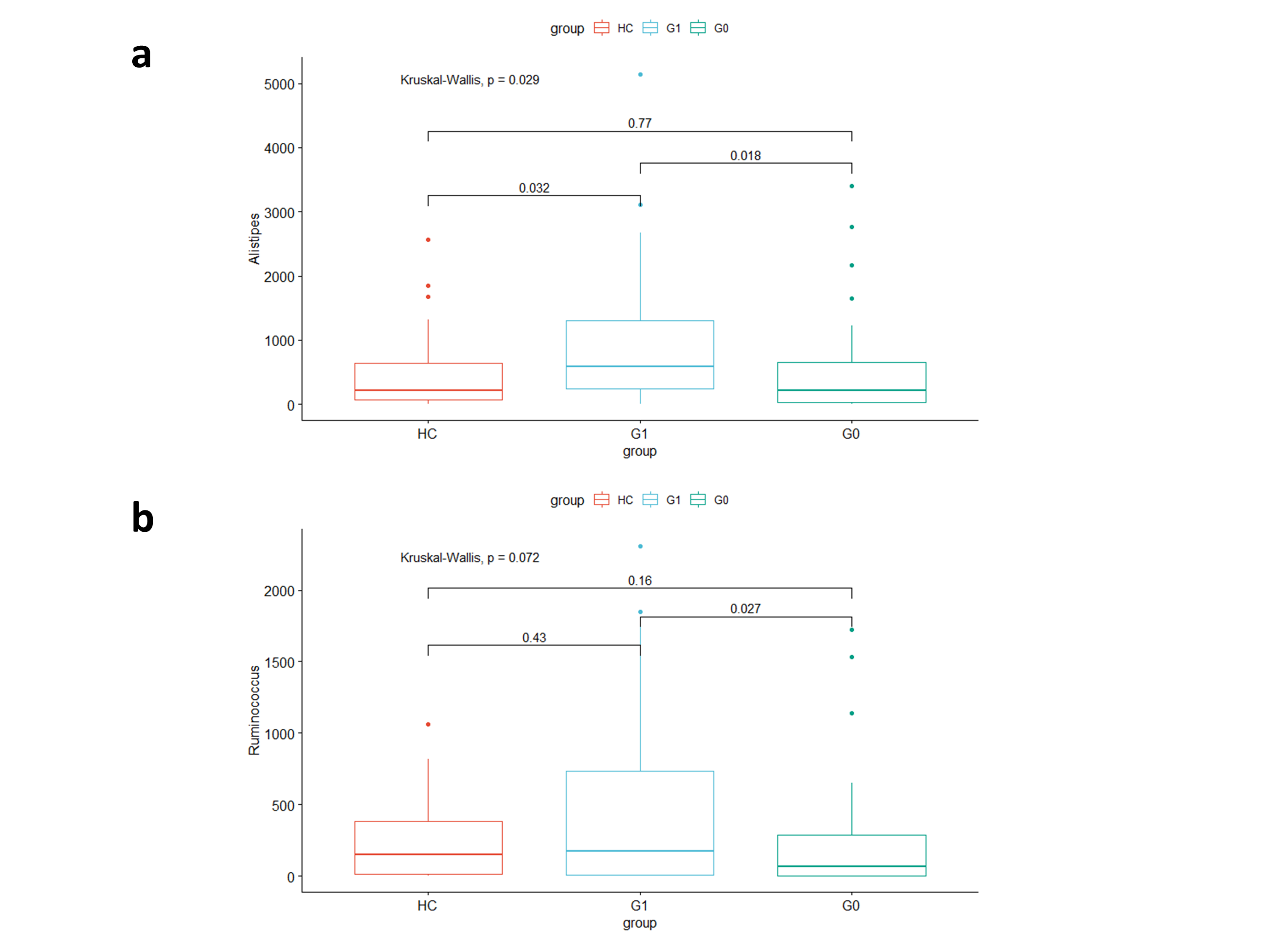


**Supplementary Figure S2. Altered gut microbiota in the GADA+ patients compared with the GADA-.** Wilcoxon test of genus differences between healthy control and T1D groups, *P < 0.05.


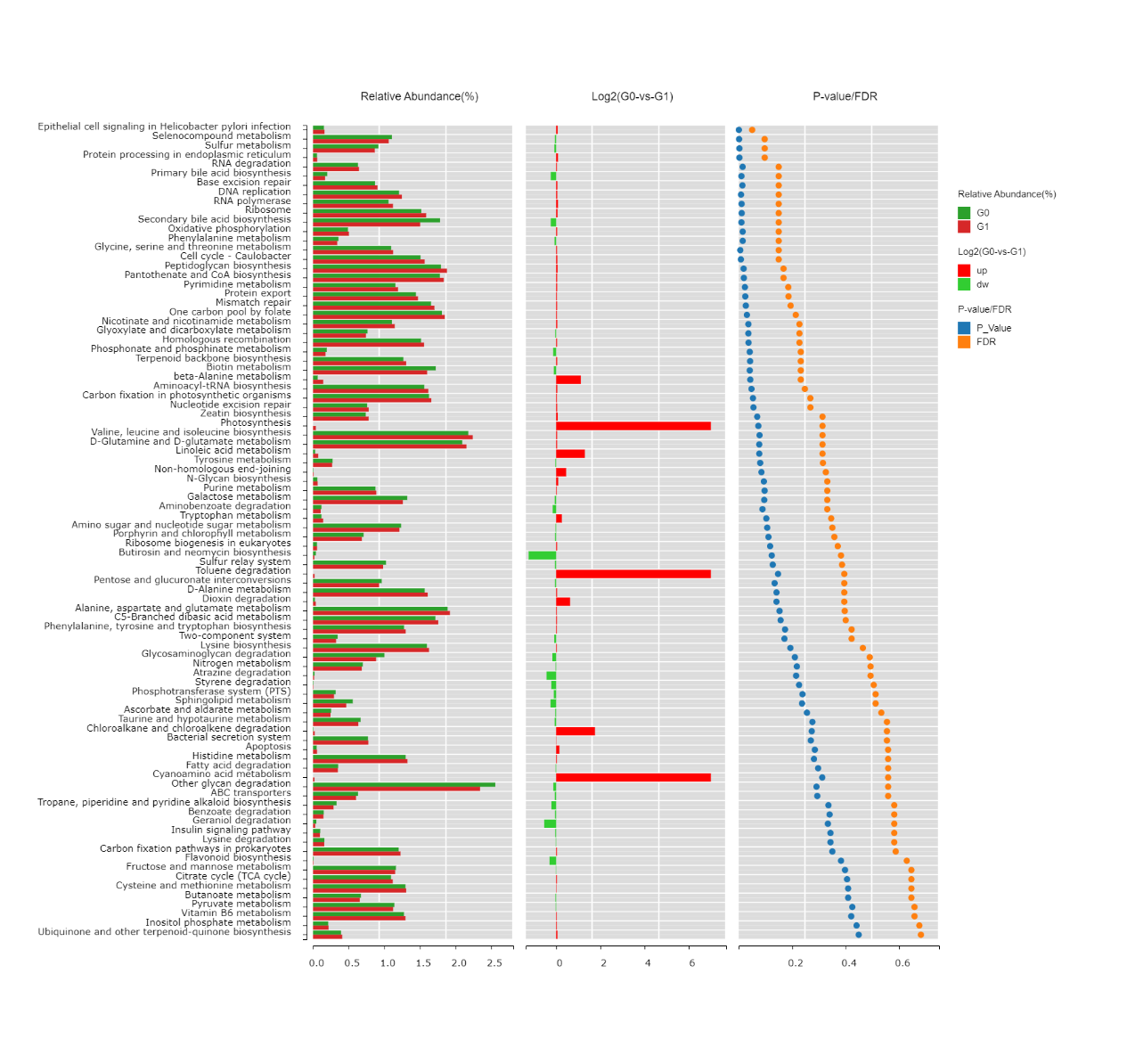


**Supplementary Figure S3. Differences in the relative abundance of the KEGG catalogs at level C and pathways.** A histogram of relative abundance for each group is shown on the left. The middle panel is the log2 value of the mean relative abundance ratio of the same pathway in the two groups. The right panel shows p-value and FDR values obtained by the Wilcoxon test, *P < 0.05.


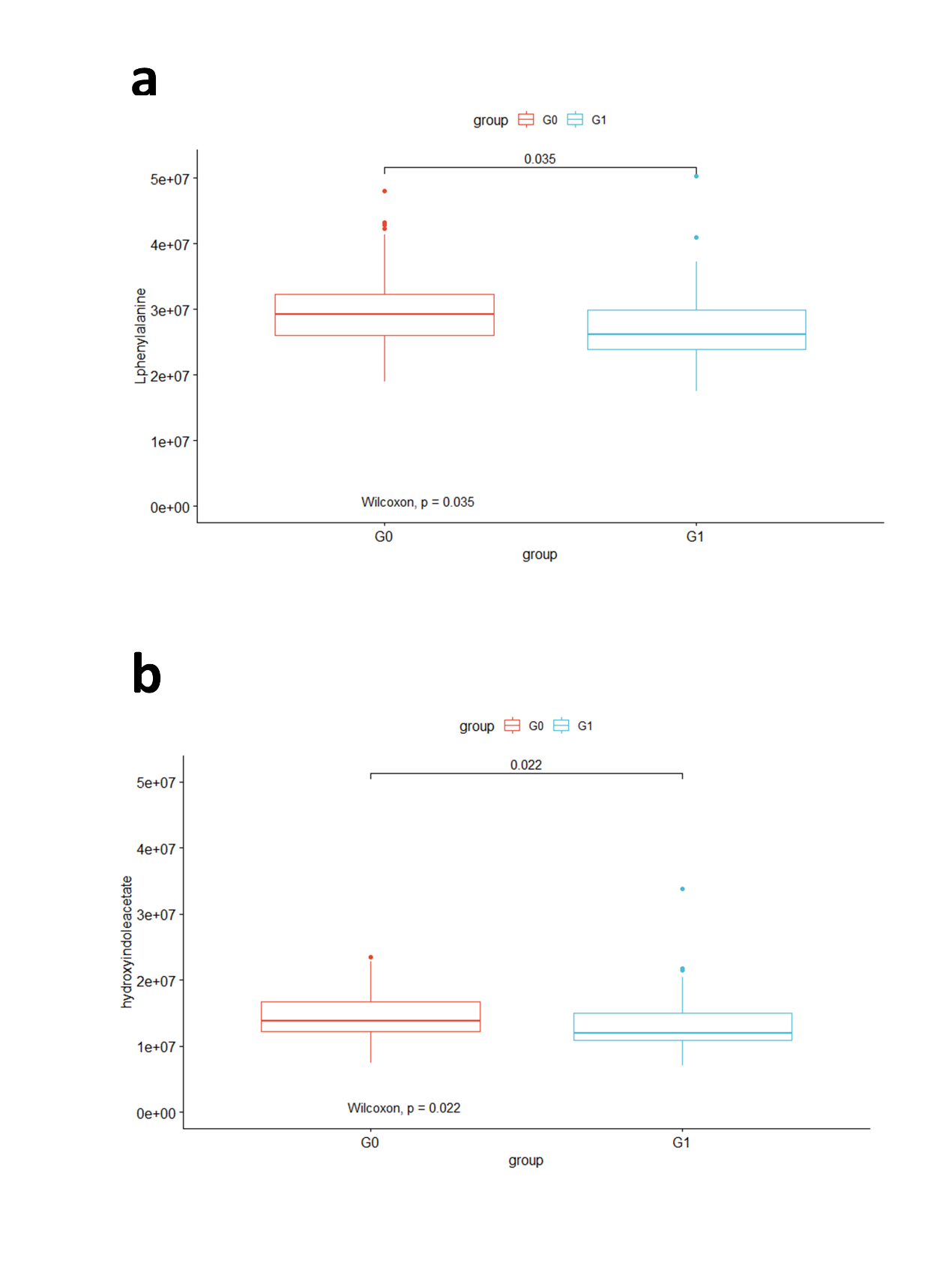


**Supplementary Figure S4. Altered serum metabolites in the GADA+ patients compared with the GADA-.** Wilcoxon test of L-phenylalanine and 5-hydroxyindoleacetate between GADA+ and GADA- patients, *P < 0.05

**2 Supplementary Tables**

## Supplementary Table S1. Clinical characteristics of the sample donors. (G0: GADA-; G1: GADA+; HC: healthy controls)

| **Number** | **group** | **Age** | **Sex** | **BMI** | **HbA1c** | **CHOL** | **TRIG** | **HDLC** | **LDLC** | **Smoking** | **titers** |
| --- | --- | --- | --- | --- | --- | --- | --- | --- | --- | --- | --- |
| HC | HC | 33 | 2 | 24.80 | 5.8 | 6.00 | 0.88 | 1.38 | 4.00 | 0 | NA |
| HC | HC | 25 | 2 | 19.68 | 5.3 | 4.30 | 0.89 | 1.29 | 2.29 | 0 | NA |
| HC | HC | 49 | 2 | 23.22 | 5.4 | 5.57 | 1.46 | 1.18 | 3.50 | 0 | NA |
| HC | HC | 42 | 2 | 19.91 | 5.4 | 4.49 | 0.70 | 1.68 | 2.27 | 0 | NA |
| HC | HC | 26 | 2 | 19.49 | 4.9 | 3.84 | 0.44 | 1.41 | 2.06 | 0 | NA |
| HC | HC | 26 | 2 | 18.86 | 4.8 | 4.52 | 1.07 | 1.25 | 2.79 | 0 | NA |
| HC | HC | 37 | 2 | 21.88 | 5.6 | 5.70 | 1.63 | 1.12 | 3.84 | 0 | NA |
| HC | HC | 12 | 2 | 19.47 | 5.4 | 5.65 | 1.02 | 1.55 | 3.45 | 0 | NA |
| HC | HC | 16 | 2 | 20.08 | 5.0 | 4.31 | 0.76 | 1.62 | 2.22 | 0 | NA |
| HC | HC | 6 | 2 | 15.45 | 5.3 | 6.33 | 0.73 | 1.55 | 3.97 | 0 | NA |
| HC | HC | 28 | 2 | 20.70 | 5.1 | 4.85 | 0.48 | 1.79 | 2.41 | 0 | NA |
| HC | HC | 34 | 1 | 26.87 | 4.9 | 5.38 | 1.76 | 1.18 | 3.31 | 1 | NA |
| HC | HC | 27 | 2 | 27.87 | 5.3 | 4.49 | 0.73 | 1.03 | 3.08 | 0· | NA |
| HC | HC | 43 | 1 | 30.12 | 5.0 | 4.43 | 1.43 | 0.84 | 2.85 | 1 | NA |
| HC | HC | 38 | 1 | 24.21 | 5.4 | 5.52 | 1.64 | 1.22 | 3.49 | 1 | NA |
| HC | HC | 31 | 2 | 22.41 | 5.4 | 3.73 | 0.88 | 1.09 | 2.30 | 0 | NA |
| HC | HC | 43 | 1 | 23.11 | 5.5 | 4.25 | 2.02 | 1.09 | 2.26 | 1 | NA |
| HC | HC | 26 | 1 | 19.47 | 5.0 | 3.55 | 0.47 | 1.23 | 1.90 | 0 | NA |
| HC | HC | 28 | 1 | 22.76 | 5.5 | 6.10 | 0.86 | 1.08 | 4.42 | 1 | NA |
| HC | HC | 30 | 2 | 22.31 | 5.2 | 3.78 | 0.50 | 1.49 | 1.88 | 0 | NA |
| HC | HC | 23 | 2 | 20.20 | 5.2 | 4.77 | 0.57 | 1.80 | 2.29 | 0 | NA |
| HC | HC | 26 | 2 | 20.55 | 4.3 | 4.58 | 0.84 | 1.20 | 2.71 | 1 | NA |
| HC | HC | 27 | 2 | 22.64 | 5.5 | 5.84 | 0.58 | 1.28 | 4.06 | 0 | NA |
| HC | HC | 26 | 2 | 16.80 | 4.9 | 5.20 | 0.96 | 1.53 | 2.95 | 0 | NA |
| HC | HC | 9 | 1 | 16.07 | 5.5 | 4.16 | 0.77 | 1.18 | 2.49 | 0 | NA |
| HC | HC | 25 | 2 | 19.88 | 5.2 | 5.15 | 0.63 | 1.61 | 3.01 | 0 | NA |
| HC | HC | 30 | 2 | 19.29 | 5.1 | 4.87 | 0.82 | 1.60 | 2.65 | 0 | NA |
| HC | HC | 25 | 2 | 22.31 | 5.3 | 4.42 | 0.51 | 1.22 | 2.75 | 0 | NA |
| HC | HC | 16 | 2 | 24.43 | 5.6 | 3.11 | 0.70 | 1.53 | 1.09 | 0 | NA |
| HC | HC | 17 | 1 | 18.50 | 5.6 | 4.01 | 1.32 | 0.94 | 2.61 | 0 | NA |
| HC | HC | 40 | 2 | 22.27 | 5.1 | 4.98 | 0.50 | 1.29 | 3.06 | 0 | NA |
| HC | HC | 25 | 1 | 18.34 | 5.2 | 3.78 | 1.02 | 1.02 | 2.43 | 0 | NA |
| HC | HC | 8 | 1 | 17.86 | 5.6 | 4.68 | 0.98 | 1.21 | 2.84 | 0 | NA |
| HC | HC | 5 | 2 | 31.40 | 5.0 | 3.76 | 0.51 | 1.30 | 2.01 | 0 | NA |
| HC | HC | 28 | 1 | 23.11 | 4.7 | 4.07 | 0.79 | 1.31 | 2.29 | 0 | NA |
| HC | HC | 53 | 1 | 23.30 | 5.7 | 5.30 | 2.86 | 1.04 | 2.88 | 1 | NA |
| HC | HC | 27 | 1 | 20.96 | 5.0 | 3.93 | 1.04 | 0.99 | 2.47 | 0 | NA |
| HC | HC | 17 | 1 | 19.44 | 5.4 | 3.86 | 0.85 | 0.98 | 2.59 | 0 | NA |
| T1 | 0 | 17 | 1 | 18.06 | 7.4 | 3.59 | 0.35 | 1.35 | 2.00 | 0 | 0.0346 |
| T10 | 0 | 15 | 1 | 18.70748 | 8.1 | 3.74 | 0.79 | 1.22 | 2.28 | 0 | 0.0023 |
| T100 | 0 | 32 | 2 | 21.30302 | 8.4 | 3.00 | 0.51 | 1.12 | 1.65 | 0 | -0.0002 |
| T101 | 0 | 29 | 1 | 20.72144 | 6.7 | 4.83 | 0.68 | 1.51 | 2.99 | 0 | 0.0295 |
| T11 | 1 | 11 | 2 | 18.64964 | 7.1 | 6.26 | 0.46 | 2.10 | 3.69 | 0 | 0.2206 |
| T12 | 1 | 9 | 2 | 13.28502 | 7.0 | 3.73 | 0.69 | 1.22 | 2.22 | 0 | 0.0740 |
| T13 | 1 | 39 | 2 | 21.52695 | 7.3 | 3.41 | 0.61 | 1.34 | 1.84 | 0 | 0.8031 |
| T14 | 0 | 40 | 2 | 20.92 | 7.5 | 5.10 | 0.69 | 1.63 | 3.17 | 0 | 0.0173 |
| T15 | 0 | 31 | 1 | 23.42 | 5.8 | 4.66 | 0.92 | 1.40 | 2.79 | 0 | -0.0117 |
| T16 | 0 | 9 | 1 | 19.38 | 7.9 | 4.82 | 1.00 | 1.54 | 2.89 | 0 | 0.0075 |
| T17 | 1 | 10 | 1 | 15.42363 | 9.1 | 4.93 | 0.49 | 1.48 | 3.27 | 0 | 0.3704 |
| T18 | 1 | 25 | 2 | 20.85328 | 7.4 | 4.21 | 0.67 | 1.55 | 2.40 | 0 | 0.2776 |
| T19 | 1 | 37 | 2 | 21.17188 | 6.4 | 5.17 | 1.17 | 1.42 | 3.36 | 0 | 0.2324 |
| T2 | 1 | 24 | 2 | 22.83 | 9.0 | 5.72 | 0.76 | 1.71 | 3.67 | 0 | 0.9191 |
| T20 | 1 | 54 | 2 | 17.26563 | 7.2 | 5.75 | 0.69 | 2.15 | 3.02 | 0 | 0.0720 |
| T21 | 0 | 15 | 1 | 16.47 | 8.8 | 4.19 | 0.54 | 1.56 | 2.20 | 0 | 0.0134 |
| T22 | 0 | 13 | 1 | 17.73 | 9.7 | 3.78 | 1.32 | 1.15 | 1.31 | 0 | -0.0119 |
| T23 | 1 | 9 | 1 | 16.44858 | 6.9 | 4.08 | 0.69 | 1.72 | 2.07 | 0 | 1.0002 |
| T24 | 0 | 39 | 2 | 20.65 | 6.2 | 5.43 | 0.50 | 2.07 | 3.16 | 0 | -0.0057 |
| T25 | 0 | 34 | 1 | 22.14 | 6.1 | 5.25 | 0.58 | 1.72 | 3.26 | 0 | 0.0331 |
| T26 | 1 | 32 | 1 | 22.95918 | 8.6 | 5.20 | 0.66 | 1.29 | 3.85 | 0 | 0.3184 |
| T27 | 1 | 20 | 2 | 19.04 | 7.5 | 4.45 | 0.85 | 1.10 | 3.15 | 0 | 0.5332 |
| T28 | 0 | 27 | 2 | 24.61515 | 8.6 | 3.82 | 0.68 | 1.34 | 2.21 | 0 | 0.0090 |
| T29 | 1 | 10 | 2 | 14.91 | 7.8 | 4.24 | 0.75 | 1.66 | 2.36 | 0 | 1.4016 |
| T3 | 0 | 17 | 2 | 21.81 | 8.1 | 4.71 | 0.58 | 1.50 | 2.90 | 0 | 0.0004 |
| T30 | 1 | 18 | 1 | 24.35306 | 9.1 | 3.83 | 0.46 | 1.21 | 2.42 | 0 | 1.6093 |
| T31 | 0 | 9 | 1 | 14.70073 | 8.3 | 3.85 | 0.59 | 1.32 | 2.41 | 0 | 0.0091 |
| T32 | 1 | 24 | 2 | 19.34 | 8.2 | 4.56 | 0.44 | 1.29 | 2.87 | 0 | 0.2135 |
| T33 | 0 | 54 | 1 | 20.46 | 5.0 | 4.28 | 0.86 | 1.43 | 2.25 | 0 | 0.0179 |
| T34 | 1 | 31 | 2 | 23.07 | 5.8 | 5.65 | 0.79 | 1.15 | 4.25 | 0 | 0.7204 |
| T35 | 1 | 29 | 1 | 23.23766 | 8.2 | 3.85 | 0.42 | 1.39 | 2.08 | 0 | 1.1127 |
| T36 | 0 | 33 | 2 | 19.62 | 6.4 | 4.45 | 0.47 | 1.75 | 2.01 | 0 | 0.0035 |
| T37 | 1 | 38 | 2 | 18.26 | 7.7 | 4.73 | 0.44 | 1.98 | 2.13 | 0 | 0.2075 |
| T38 | 1 | 13 | 2 | 21.46814 | 12.1 | 4.67 | 0.51 | 1.81 | 2.36 | 0 | 0.0696 |
| T39 | 1 | 10 | 2 | 17.26133 | 5.8 | 4.26 | 0.67 | 1.56 | 2.23 | 0 | 0.2223 |
| T4 | 1 | 11 | 2 | 18.73767 | 6.6 | 3.96 | 0.82 | 1.72 | 1.74 | 0 | 0.1515 |
| T40 | 1 | 26 | 2 | 25.10 | 8.5 | 3.82 | 0.59 | 1.60 | 2.88 | 0 | 0.2589 |
| T41 | 1 | 24 | 1 | 17.49 | 7.2 | 4.18 | 0.52 | 1.39 | 2.85 | 0 | 0.1424 |
| T42 | 1 | 17 | 1 | 18.44 | 8.4 | 4.24 | 0.73 | 1.36 | 2.68 | 0 | 0.1643 |
| T43 | 1 | 8 | 2 | 16.31021 | 10.7 | 3.80 | 0.34 | 1.57 | 1.92 | 0 | 0.3962 |
| T44 | 1 | 28 | 1 | 21.07266 | 7.3 | 5.04 | 0.64 | 1.70 | 2.96 | 1 | 0.0652 |
| T45 | 1 | 30 | 2 | 23.24263 | 6.6 | 5.33 | 0.94 | 1.68 | 3.17 | 0 | 1.0272 |
| T46 | 1 | 27 | 2 | 19.49219 | 7.5 | 4.49 | 0.66 | 1.63 | 2.64 | 0 | 0.5891 |
| T47 | 0 | 41 | 1 | 20.93 | 5.3 | 5.17 | 1.10 | 1.27 | 3.27 | 1 | 0.0297 |
| T48 | 0 | 24 | 1 | 24.7586 | 7.1 | 5.16 | 0.87 | 1.33 | 3.41 | 1 | -0.0084 |
| T49 | 0 | 32 | 1 | 21.75 | 7.0 | 5.17 | 0.52 | 1.61 | 3.21 | 0 | 0.0036 |
| T5 | 1 | 13 | 2 | 21.80071 | 8.1 | 6.83 | 0.76 | 1.77 | 4.64 | 0 | 0.9741 |
| T50 | 0 | 20 | 1 | 21.45 | 7.9 | 4.19 | 0.48 | 1.54 | 2.37 | 0 | 0.0255 |
| T51 | 0 | 18 | 1 | 18.08 | 10.1 | 5.89 | 0.48 | 0.95 | 4.76 | 0 | 0.0312 |
| T52 | 1 | 17 | 2 | 21.97551 | 7.8 | 4.42 | 0.96 | 1.53 | 2.35 | 0 | 0.0691 |
| T53 | 1 | 14 | 1 | 18.04623 | 8.1 | 4.58 | 0.49 | 1.61 | 2.67 | 0 | 0.0471 |
| T54 | 1 | 15 | 2 | 23.10868 | 11.8 | 4.60 | 0.56 | 1.46 | 2.93 | 0 | 0.5180 |
| T55 | 0 | 30 | 2 | 20.89334 | 6.1 | 4.04 | 0.94 | 1.51 | 2.11 | 0 | 0.0168 |
| T56 | 1 | 21 | 1 | 18.82813 | 10.5 | 4.89 | 0.55 | 1.35 | 3.36 | 1 | 0.2561 |
| T57 | 0 | 32 | 2 | 20.23438 | 6.2 | 5.01 | 0.71 | 1.55 | 3.20 | 0 | 0.0180 |
| T58 | 0 | 12 | 2 | 21.32813 | 14.7 | 3.57 | 0.75 | 1.36 | 1.79 | 0 | -0.0035 |
| T59 | 0 | 36 | 1 | 22.04082 | 8.2 | 4.41 | 1.09 | 1.44 | 2.60 | 1 | 0.0091 |
| T6 | 1 | 11 | 1 | 15.55556 | 5.8 | 5.25 | 0.65 | 1.81 | 2.91 | 0 | 2.0241 |
| T60 | 1 | 9 | 1 | 19.53361 | 7.6 | 4.52 | 0.59 | 1.89 | 2.11 | 0 | 0.1692 |
| T61 | 1 | 21 | 1 | 17.59412 | 8.7 | 4.26 | 0.51 | 1.63 | 2.15 | 1 | 1.6431 |
| T62 | 0 | 47 | 1 | 19.21214 | 7.4 | 4.78 | 0.83 | 1.50 | 2.86 | 1 | 0.0078 |
| T63 | 1 | 21 | 2 | 17.99184 | 7.4 | 4.19 | 0.62 | 1.51 | 2.32 | 0 | 1.6996 |
| T64 | 0 | 28 | 1 | 18.13131 | 7.5 | 4.72 | 0.61 | 2.10 | 2.79 | 0 | 0.0097 |
| T65 | 0 | 25 | 2 | 21.25 | 7.6 | 5.45 | 0.81 | 1.75 | 3.39 | 0 | 0.0319 |
| T66 | 1 | 45 | 2 | 22.63971 | 7.5 | 5.11 | 0.50 | 1.86 | 2.84 | 0 | 0.6453 |
| T67 | 0 | 40 | 1 | 20.08 | 6.3 | 4.46 | 1.90 | 1.10 | 3.09 | 1 | -0.0397 |
| T68 | 1 | 37 | 2 | 21.13281 | 6.2 | 5.01 | 0.64 | 1.73 | 2.75 | 0 | 0.1794 |
| T69 | 0 | 41 | 2 | 24.0172 | 8.1 | 4.86 | 0.58 | 1.64 | 2.73 | 0 | -0.0111 |
| T7 | 0 | 10 | 2 | 17.27367 | 7.2 | 3.87 | 1.12 | 1.90 | 1.51 | 0 | 0.0239 |
| T70 | 0 | 34 | 2 | 22.11 | 6.3 | 3.00 | 0.50 | 1.39 | 1.22 | 0 | -0.0110 |
| T71 | 0 | 33 | 2 | 21.64824 | 6.9 | 3.64 | 0.61 | 1.37 | 1.79 | 0 | -0.0182 |
| T72 | 1 | 26 | 2 | 20.0551 | 5.6 | 5.33 | 0.68 | 1.79 | 2.84 | 0 | 1.0610 |
| T73 | 1 | 29 | 2 | 21.35 | 5.8 | 4.57 | 0.70 | 1.79 | 2.25 | 0 | 0.1462 |
| T74 | 1 | 31 | 2 | 22.67 | 6.2 | 4.15 | 0.49 | 1.16 | 2.75 | 0 | 0.8828 |
| T75 | 0 | 41 | 1 | 20.64 | 5.2 | 5.46 | 1.04 | 1.68 | 3.09 | 0 | 0.0373 |
| T76 | 1 | 26 | 2 | 21.40861 | 6.0 | 4.33 | 0.50 | 1.57 | 2.38 | 0 | 0.0496 |
| T77 | 0 | 29 | 2 | 18.67093 | 6.6 | 6.54 | 0.53 | 2.59 | 2.87 | 0 | -0.0016 |
| T78 | 0 | 35 | 2 | 20.87005 | 6.0 | 5.19 | 0.64 | 1.48 | 3.22 | 0 | 0.0090 |
| T79 | 0 | 38 | 2 | 20.58594 | 5.7 | 4.32 | 0.98 | 1.62 | 2.27 | 0 | -0.0016 |
| T8 | 0 | 11 | 2 | 15.86987 | 13.8 | 6.05 | 1.61 | 1.57 | 4.02 | 0 | -0.0047 |
| T80 | 0 | 44 | 1 | 22.11 | 6.5 | 5.39 | 0.58 | 1.69 | 3.31 | 0 | -0.0095 |
| T81 | 0 | 24 | 2 | 21.49 | 7.0 | 4.69 | 0.65 | 1.33 | 3.01 | 0 | 0.0092 |
| T82 | 1 | 39 | 2 | 22.70 | 7.2 | 4.06 | 0.71 | 1.34 | 2.48 | 0 | 0.3594 |
| T83 | 0 | 26 | 2 | 18.09 | 5.8 | 3.99 | 0.49 | 1.49 | 1.94 | 0 | 0.0087 |
| T84 | 0 | 29 | 2 | 22.18279 | 5.1 | 5.66 | 0.68 | 1.86 | 3.11 | 0 | 0.0317 |
| T85 | 1 | 39 | 2 | 22.59253 | 6.7 | 6.92 | 0.53 | 1.79 | 4.55 | 0 | 1.2239 |
| T86 | 1 | 45 | 1 | 23.35183 | 7.0 | 6.51 | 2.22 | 1.16 | 4.26 | 0 | 0.2238 |
| T87 | 0 | 46 | 1 | 19.38 | 6.7 | 4.16 | 1.28 | 1.31 | 2.06 | 0 | -0.0092 |
| T88 | 0 | 34 | 2 | 25.67697 | 9.4 | 6.43 | 1.62 | 2.25 | 3.34 | 0 | -0.0004 |
| T89 | 0 | 31 | 1 | 22.58271 | 13.1 | 6.23 | 0.93 | 1.16 | 4.57 | 0 | 0.0071 |
| T9 | 0 | 10 | 2 | 16.54915 | 10.6 | 3.88 | 0.72 | 1.24 | 2.26 | 0 | 0.0019 |
| T90 | 1 | 44 | 1 | 19.74721 | 7.8 | 4.87 | 1.19 | 1.51 | 2.97 | 1 | 0.3237 |
| T91 | 0 | 34 | 2 | 22.96 | 6.7 | 3.97 | 0.93 | 1.31 | 2.14 | 0 | -0.0056 |
| T92 | 0 | 30 | 2 | 19.66 | 5.9 | 4.62 | 0.79 | 1.58 | 2.64 | 0 | 0.0053 |
| T93 | 0 | 34 | 1 | 24.50 | 8.2 | 5.27 | 1.16 | 1.09 | 3.80 | 1 | -0.0063 |
| T94 | 1 | 42 | 2 | 25.38371 | 7.1 | 5.67 | 1.06 | 1.78 | 3.45 | 0 | 0.0833 |
| T95 | 1 | 40 | 1 | 23.03 | 7.2 | 5.10 | 0.60 | 1.73 | 3.06 | 0 | 0.2715 |
| T96 | 0 | 39 | 1 | 20.98 | 6.5 | 3.33 | 1.58 | 0.80 | 1.89 | 0 | 0.0035 |
| T97 | 1 | 11 | 1 | 16.33579 | 7.6 | 3.98 | 0.55 | 1.30 | 2.32 | 0 | 0.8784 |
| T98 | 0 | 29 | 2 | 21.01977 | 7.1 | 4.24 | 0.53 | 1.64 | 2.14 | 0 | -0.0080 |
| T99 | 0 | 21 | 2 | 21.83391 | 11.1 | 6.70 | 1.18 | 1.83 | 4.39 | 0 | 0.0264 |

## Supplementary Table S2. The relative abundance of taxonomic profiling on 16s sequencing data of fecal samples. The abundant taxa (phylum level) were shown. G0, GADA-; G1, GADA+; HC, healthy controls.

| phylum | G0 | G0% | G1 | G1% | HC | HC % |
| --- | --- | --- | --- | --- | --- | --- |
| Actinobacteriota | 10751 | 0.55 | 4131 | 0.25 | 4691 | 0.32 |
| Bacteroidota | 1221171 | 62.42 | 1034599 | 62.48 | 942623 | 63.81 |
| Desulfobacterota | 6490 | 0.33 | 3879 | 0.23 | 5452 | 0.37 |
| Firmicutes | 577748 | 29.53 | 505305 | 30.52 | 430625 | 29.15 |
| Proteobacteria | 140341 | 7.17 | 107888 | 6.52 | 93811 | 6.35 |

**Supplementary Table S3. The LEFse analysis constructed based on the genus level.**

| Genus | group | LDA-score | Pvalues |
| --- | --- | --- | --- |
| Bacteria.Firmicutes.Clostridia.Clostridiales.Ruminococcaceae | G1 | 4.077156565 | 0.045745259 |
| Bacteria.Bacteroidetes.Bacteroidia.Bacteroidales.Rikenellaceae.Alistipes | G1 | 3.802602544 | 0.029408907 |
| Bacteria.Bacteroidetes.Bacteroidia.Bacteroidales.Rikenellaceae | G1 | 3.802602544 | 0.029408907 |
| Bacteria.Firmicutes.Clostridia.Clostridiales.Ruminococcaceae.Ruminococcus | G1 | 3.770642266 | 0.044302314 |
| Bacteria.Firmicutes.Negativicutes.Selenomonadales.Veillonellaceae.Dialister | G1 | 3.390634231 | 0.01249824 |
| Bacteria.Bacteroidetes.Bacteroidia.Bacteroidales.Porphyromonadaceae.Coprobacter | G1 | 3.263984456 | 0.020216688 |
| Bacteria.Lentisphaerae.Lentisphaeria.Victivallales.Victivallaceae | G1 | 3.241093037 | 0.031323041 |
| Bacteria.Firmicutes.Clostridia.Clostridiales.Eubacteriaceae | G1 | 3.125831649 | 0.01078359 |
| Bacteria.Firmicutes.Clostridia.Clostridiales.Eubacteriaceae.Eubacterium | G1 | 3.125377964 | 0.014007635 |
| Bacteria.Lentisphaerae.Lentisphaeria | G1 | 3.115805887 | 0.031323041 |
| Bacteria.Lentisphaerae.Lentisphaeria.Victivallales.Victivallaceae.Victivallis | G1 | 3.105558573 | 0.031323041 |
| Bacteria.Lentisphaerae.Lentisphaeria.Victivallales | G1 | 3.088286285 | 0.031323041 |
| Bacteria.Firmicutes.Bacilli.Lactobacillales.Enterococcaceae | G1 | 3.064041727 | 0.002544315 |
| Bacteria.Lentisphaerae | G1 | 3.052411227 | 0.031323041 |
| Bacteria.Firmicutes.Bacilli.Lactobacillales.Enterococcaceae.Enterococcus | G1 | 3.040365666 | 0.002544315 |
| Bacteria.Proteobacteria.Betaproteobacteria.Burkholderiales.Oxalobacteraceae | G1 | 2.419502803 | 0.022885877 |
| Bacteria.Proteobacteria.Betaproteobacteria.Burkholderiales.Oxalobacteraceae.Oxalobacter | G1 | 2.413588797 | 0.022885877 |
| Bacteria.Firmicutes.Clostridia.Clostridiales.Clostridiales | G1 | 2.343182343 | 0.047105165 |
| Bacteria.Firmicutes.Clostridia.Clostridiales.Ruminococcaceae.Intestinimonas | G1 | 2.2177778 | 0.012514634 |
| Bacteria.Firmicutes.Clostridia.Clostridiales.Clostridiales.Mogibacterium | G1 | 2.203260116 | 0.014465502 |
| Bacteria | G0 | 3.548953259 | 1.06273E-07 |
| Bacteria.Firmicutes.Erysipelotrichia.Erysipelotrichales.Erysipelotrichaceae.Clostridium_XVIII | G0 | 2.757993012 | 0.037389622 |

**Supplementary Table S4. KEGG functional predictions were generated using the PICRUSt at level B&C and pathways.** Wilcoxon test of pathway differences between GADA+ and GADA- groups. P values and false discovery rate values obtained by the Wilcoxon test, the table shows P < 0.05.

| function_levelB | G0(%) | G1(%) | p value | FDR |
| --- | --- | --- | --- | --- |
| Replication and repair | 6.014738 | 6.203989 | 0.009534 | 0.087488 |
| Cell growth and death | 1.559843 | 1.622557 | 0.010725 | 0.087488 |
| Transcription | 1.059323 | 1.12263 | 0.010936 | 0.087488 |
| Lipid metabolism | 6.188485 | 5.818556 | 0.010936 | 0.087488 |
| Translation | 3.19031 | 3.316428 | 0.022224 | 0.142234 |
| Nucleotide metabolism | 2.032737 | 2.081868 | 0.040492 | 0.204119 |
| Amino acid metabolism | 12.708943 | 12.907126 | 0.044651 | 0.204119 |
| Biosynthesis of other secondary metabolites | 2.338733 | 2.264604 | 0.07671 | 0.30684 |

| function_levelC | G0(%) | G1(%) | p value | FDR |
| --- | --- | --- | --- | --- |
| Selenocompound metabolism | 1.10678 | 1.06221 | 0.001204 | 0.096922 |
| Sulfur metabolism | 0.918718 | 0.866845 | 0.00236 | 0.097163 |
| Protein processing in endoplasmic reticulum | 0.055083 | 0.05839 | 0.002414 | 0.097163 |
| Glycine, serine and threonine metabolism | 1.097408 | 1.125162 | 0.005742 | 0.149428 |
| Chagas disease (American trypanosomiasis) | 4.83E-4 | 1.12E-4 | 0.007324 | 0.149428 |
| Cell cycle - Caulobacter | 1.509965 | 1.567429 | 0.007967 | 0.149428 |
| DNA replication | 1.206954 | 1.249073 | 0.008462 | 0.149428 |
| Primary bile acid biosynthesis | 0.198226 | 0.167105 | 0.010114 | 0.149428 |
| Secondary bile acid biosynthesis | 1.784025 | 1.503934 | 0.010114 | 0.149428 |
| RNA polymerase | 1.059253 | 1.122577 | 0.010725 | 0.149428 |
| Ribosome | 1.519586 | 1.587627 | 0.011151 | 0.149428 |
| Base excision repair | 0.869495 | 0.903686 | 0.01377 | 0.149428 |
| RNA degradation | 0.630581 | 0.644015 | 0.014573 | 0.149428 |
| Oxidative phosphorylation | 0.489162 | 0.503886 | 0.01485 | 0.149428 |
| Phenylalanine metabolism | 0.357226 | 0.338987 | 0.01485 | 0.149428 |
| Peptidoglycan biosynthesis | 1.794807 | 1.879467 | 0.018212 | 0.167499 |
| Pantothenate and CoA biosynthesis | 1.781781 | 1.836453 | 0.019238 | 0.167499 |
| Plant hormone signal transduction | 4.1E-5 | 1.9E-5 | 0.019767 | 0.167499 |
| Pyrimidine metabolism | 1.157543 | 1.193191 | 0.02303 | 0.185392 |
| Protein export | 1.444105 | 1.473232 | 0.024288 | 0.186208 |
| Mismatch repair | 1.656773 | 1.703453 | 0.026516 | 0.194049 |
| One carbon pool by folate | 1.811325 | 1.849385 | 0.030448 | 0.213136 |
| Nicotinate and nicotinamide metabolism | 1.107064 | 1.146228 | 0.036059 | 0.227047 |
| Glyoxylate and dicarboxylate metabolism | 0.76419 | 0.743342 | 0.036059 | 0.227047 |
| Homologous recombination | 1.516687 | 1.557406 | 0.036666 | 0.227047 |
| Phosphonate and phosphinate metabolism | 0.191419 | 0.174504 | 0.041161 | 0.232049 |
| Biotin metabolism | 1.724643 | 1.603 | 0.041161 | 0.232049 |
| Terpenoid backbone biosynthesis | 1.268148 | 1.308627 | 0.042528 | 0.232049 |
| beta-Alanine metabolism | 0.065358 | 0.142104 | 0.043239 | 0.232049 |
| Aminoacyl-tRNA biosynthesis | 1.560689 | 1.617391 | 0.047619 | 0.247312 |

**Supplementary Table S5. Metabolites identification in serum from T1D patients with and without GADA.**

| Class | HMDBID | KEGGID | G0.MeanSD | G1.MeanSD | G0.MedianIQR | G1.MedianIQR | FC | P | FDR.P |
| --- | --- | --- | --- | --- | --- | --- | --- | --- | --- |
| Steroids and steroid derivatives | HMDB0000384 | NA | 18.22 (28.58) | 6.19 (9.06) | 4.78 [3.137,23.575] | 2.7 [1.449,5.832] | 0.57 | 6.90E-04 | 0.579 |
| Steroids and steroid derivatives | HMDB0000308 | NA | 79.81 (139.6) | 25.62 (37.74) | 31.43 [10.137,101.71] | 9.73 [5.91,27.135] | 0.31 | 8.20E-04 | 0.579 |
| Amino acids | HMDB0000159 | C00079 | 22109.22 (7360.34) | 17671.54 (5436.88) | 21225.13 [17104.95,26696.507] | 16957.69 [13011.762,21781.765] | 0.8 | 2.10E-03 | 0.579 |
| Cinnamic acids and derivatives | HMDB0000930 | C10438 | 22816.74 (7340.98) | 18367.15 (5462.35) | 22108.59 [17730.895,27456.917] | 17495.6 [13885.105,22732.972] | 0.79 | 2.10E-03 | 0.579 |
| Steroids and steroid derivatives | HMDB0002586 | NA | 3.24 (6.41) | 1 (1.45) | 0.9 [0.35,3.292] | 0.48 [0.099,1.046] | 0.53 | 3.50E-03 | 0.579 |
| Steroids and steroid derivatives | HMDB0000384 | NA | 64.77 (48.4) | 39.74 (32.99) | 49.15 [27.806,88.322] | 30.1 [16.52,53.315] | 0.61 | 3.50E-03 | 0.579 |
| Steroids and steroid derivatives | HMDB0000308 | NA | 172.01 (119.19) | 114.22 (81.83) | 146.02 [90.755,231.029] | 97.45 [66.322,148.455] | 0.67 | 4.10E-03 | 0.579 |
| Nucleosides [Fig] | HMDB0000296 | C00299 | 90.03 (44.58) | 69.67 (25.71) | 79.95 [66.184,100.672] | 65.96 [56.182,82.064] | 0.83 | 4.20E-03 | 0.579 |
| Organooxygen compounds | HMDB0000684 | C00328 | 189.57 (60.29) | 160.57 (68.65) | 175.33 [149.049,221.577] | 141.34 [118.475,193.767] | 0.81 | 5.00E-03 | 0.579 |
| Carboxylic acids and derivatives | HMDB0000832 | NA | 7.95 (5.41) | 6.14 (6.4) | 5.71 [4.57,9.928] | 4.73 [4.136,5.744] | 0.83 | 5.50E-03 | 0.579 |
| Pyrrolidines | HMDB0002039 | C11118 | 24.93 (33.35) | 16.05 (5.76) | 17.72 [14.223,22.038] | 14.63 [12.389,18.321] | 0.83 | 6.20E-03 | 0.579 |
| Carboxylic acids and derivatives | HMDB0028744 | NA | 171.16 (75.2) | 137.03 (66.68) | 162.31 [119.601,220.463] | 112.24 [87.843,173.135] | 0.69 | 6.80E-03 | 0.579 |
| Indoles and derivatives | HMDB0000763 | C05635 | 258.75 (72.59) | 224.85 (84.8) | 242.37 [219.074,295.547] | 198.82 [171.711,265.455] | 0.82 | 8.30E-03 | 0.634 |
| Organic sulfuric acids and derivatives | HMDB0062775 | NA | 103.39 (88.12) | 69.45 (54.95) | 76.96 [50.782,122.454] | 53.37 [36.464,85.754] | 0.69 | 8.30E-03 | 0.634 |
| Tropane alkaloids | HMDB0006406 | C12448 | 11.57 (5.58) | 9.33 (4.14) | 9.88 [8.003,11.957] | 8.1 [6.976,10.595] | 0.82 | 1.10E-02 | 0.662 |
| Fatty Acyls | HMDB0007329 | NA | 124.8 (79.39) | 84.08 (46.22) | 105.54 [62.55,165.263] | 66.83 [54.893,114.572] | 0.63 | 1.10E-02 | 0.662 |
| Fatty Acyls | HMDB0007329 | NA | 579.86 (256.95) | 458.92 (193.15) | 541.81 [370.357,712.68] | 402.42 [310.973,550.755] | 0.74 | 1.10E-02 | 0.662 |
| Carboxylic acids and derivatives | HMDB0003974 | C05379 | 497.11 (146.16) | 426.18 (176.5) | 495.14 [397.147,573.346] | 392.14 [315.445,498.118] | 0.79 | 1.20E-02 | 0.714 |
| Carboximidic acids and derivatives | HMDB0041901 | NA | 1.86 (4.07) | 2.85 (5.29) | 0.49 [0.177,1.3] | 1.36 [0.352,2.895] | 2.79 | 1.30E-02 | 0.727 |
| Organooxygen compounds | HMDB0000684 | C00328 | 12.26 (3.7) | 11.05 (3.99) | 12.43 [10.195,14.247] | 10.04 [8.843,12.43] | 0.81 | 1.40E-02 | 0.727 |
| Carboxylic acids and derivatives | HMDB0034365 | C01047 | 305.23 (201.42) | 220.8 (179.76) | 295.12 [144.97,446.363] | 144.48 [99,318.735] | 0.49 | 1.40E-02 | 0.74 |
| Carboxylic acids and derivatives | HMDB0013286 | NA | 15.48 (11.3) | 11.45 (10.32) | 13.51 [6.542,19.153] | 8.5 [5.049,10.791] | 0.63 | 1.60E-02 | 0.74 |
| Steroids and steroid derivatives | HMDB0001547 | C02140 | 26.22 (10.53) | 21.82 (11.81) | 26.07 [17.599,31.619] | 20.41 [14.553,25.802] | 0.78 | 1.90E-02 | 0.776 |
| Benzene and substituted derivatives | HMDB0032796 | NA | 145.93 (130.51) | 152.82 (367.66) | 119.54 [58.467,172.527] | 60.87 [45.518,122.772] | 0.51 | 1.90E-02 | 0.776 |
| Benzene and substituted derivatives | HMDB0032796 | NA | 47.16 (41.92) | 47.34 (108.28) | 38.4 [19.754,59.122] | 23.79 [14.519,42.051] | 0.62 | 2.10E-02 | 0.797 |
| Tetrapyrroles and derivatives | HMDB0001898 | C05790 | 8.45 (10.97) | 18.89 (26.82) | 5.62 [0.395,11.504] | 9.51 [1.934,21.496] | 1.69 | 2.20E-02 | 0.797 |
| Benzene and substituted derivatives | HMDB0001476 | C00632 | 38.72 (16.47) | 31.65 (13.69) | 37.63 [27.599,46.326] | 29.65 [23.181,40.205] | 0.79 | 2.40E-02 | 0.821 |
| Indoles and derivatives | HMDB0000763 | C05635 | 277.46 (86.54) | 247.82 (92.8) | 277.26 [234.636,324.993] | 230.06 [193.638,303.759] | 0.83 | 2.60E-02 | 0.822 |
| Organooxygen compounds | HMDB0014371 | C07035 | 5.48 (4.2) | 5.45 (9.32) | 4.38 [3.466,5.868] | 3.3 [2.566,5.197] | 0.75 | 2.70E-02 | 0.822 |
| Fatty Acyls | HMDB0062452 | C17278 | 63 (41.08) | 45.32 (22.73) | 53.25 [35.983,81.712] | 43.42 [27.79,56.244] | 0.82 | 2.70E-02 | 0.822 |
| Dihydrofurans | HMDB0039786 | NA | 55.48 (44.45) | 36.41 (27.46) | 43.03 [23.277,69.301] | 28.4 [21.349,45.091] | 0.66 | 2.70E-02 | 0.822 |
| Carboxylic acids and derivatives | HMDB0028757 | NA | 80.11 (33.74) | 66.09 (33.71) | 73.42 [50.383,106.197] | 54.16 [45.265,77.004] | 0.74 | 2.90E-02 | 0.822 |
| Fatty Acyls | HMDB0007329 | NA | 452.13 (284.43) | 333.68 (199.35) | 396.65 [223.002,582.735] | 275.47 [201.866,420.261] | 0.69 | 2.90E-02 | 0.822 |
| Organic sulfuric acids and derivatives | HMDB0127980 | NA | 13.08 (16.15) | 8.02 (12.25) | 6.52 [3.34,18.326] | 4.39 [2.135,8.69] | 0.67 | 2.90E-02 | 0.822 |
| Sulfamic acid derivatives | HMDB0031340 | C02824 | 72.4 (151.36) | 95.34 (127.56) | 18.76 [2.662,50.369] | 61.46 [7.44,116.028] | 3.28 | 3.20E-02 | 0.877 |
| Benzodiazepines | HMDB0061053 | NA | 61.71 (32.43) | 51.14 (31.27) | 53.97 [41.967,81.004] | 42.88 [31.492,68.942] | 0.79 | 3.40E-02 | 0.889 |
| Carbonyl compounds | HMDB0031195 | C14743 | 17.84 (23.33) | 23.71 (74.51) | 9.41 [6.337,19.682] | 6.9 [5.294,10.509] | 0.73 | 3.50E-02 | 0.889 |
| Benzene and substituted derivatives | HMDB0032575 | NA | 30.12 (27.57) | 31.86 (78.73) | 23.69 [12.072,37.931] | 13.21 [9.092,24.956] | 0.56 | 3.50E-02 | 0.889 |
| Alkaloids derived from lysine | HMDB0000716 | C00408 | 11.24 (12.82) | 10.9 (19.48) | 7.49 [6.072,9.7] | 5.93 [3.496,7.854] | 0.79 | 3.70E-02 | 0.896 |
| Fatty Acyls | HMDB0007271 | NA | 1242.69 (578.27) | 1022.65 (542.48) | 1089.68 [812.858,1629.174] | 895.03 [655.421,1387.101] | 0.82 | 3.70E-02 | 0.896 |
| Glycerophospholipids | HMDB0011504 | NA | 16.98 (14.87) | 20.05 (11.19) | 13.56 [7.298,20.403] | 19.66 [9.952,27.012] | 1.45 | 3.70E-02 | 0.896 |
| Benzothiazines | HMDB0015474 | C07192 | 15.2 (26.26) | 20.19 (22.45) | 7.81 [1.667,18.63] | 12.38 [3.528,30.557] | 1.59 | 3.80E-02 | 0.896 |
| Steroids and steroid derivatives | HMDB0015148 | NA | 1.62 (10.66) | 0.16 (0.4) | 0.1 [0.044,0.206] | 0.05 [0.037,0.105] | 0.48 | 3.90E-02 | 0.896 |
| Quinolines and derivatives | HMDB0033731 | C06413 | 386.02 (188.93) | 315.78 (130.83) | 356 [258.746,466.797] | 279.73 [206.021,387.152] | 0.79 | 4.00E-02 | 0.896 |
| FA01 Fatty Acids and Conjugates | HMDB0004705 | C14829 | 891.44 (390.99) | 735.18 (309.49) | 851.68 [600.382,1174.189] | 699.66 [520.908,890.697] | 0.82 | 4.30E-02 | 0.896 |
| Fatty Acyls | HMDB0040909 | NA | 69.58 (44.48) | 51.02 (26.95) | 61.14 [35.972,89.153] | 48.72 [30.425,58.402] | 0.8 | 4.30E-02 | 0.896 |
| Fatty Acyls | HMDB0031091 | NA | 21.34 (12.41) | 17.43 (12.16) | 18.23 [13.729,26.571] | 15.16 [11.912,18.261] | 0.83 | 4.30E-02 | 0.896 |
| Imidazopyrimidines | HMDB0061068 | NA | 1.98 (1.87) | 2.03 (4.9) | 1.5 [0.716,2.446] | 0.8 [0.507,1.573] | 0.53 | 4.30E-02 | 0.896 |
| Benzopyrans | HMDB0029467 | NA | 3.11 (12.99) | 2.2 (2.64) | 0.91 [0.548,1.481] | 1.52 [0.838,2.03] | 1.67 | 4.40E-02 | 0.896 |
| Purine nucleotides | HMDB0060418 | C16618 | 26.7 (15.2) | 22.37 (15.66) | 24.53 [17.097,32.881] | 17.79 [12.859,29.147] | 0.73 | 4.50E-02 | 0.896 |
| Fatty Acyls | HMDB0002366 | NA | 37.53 (17.73) | 31.46 (12.91) | 34.64 [27.551,40.602] | 28.78 [22.36,38.953] | 0.83 | 4.50E-02 | 0.896 |
| Organic sulfuric acids and derivatives | HMDB0060013 | NA | 37.28 (82.17) | 54.16 (96.58) | 5.4 [0.873,15.863] | 9.48 [3.742,42.269] | 1.76 | 4.50E-02 | 0.896 |
| Fatty Acyls | HMDB0007329 | NA | 48.12 (30.3) | 36.03 (19.6) | 44.86 [25.299,61.942] | 28.28 [21.768,49.295] | 0.63 | 4.60E-02 | 0.896 |
| Alkaloids derived from tryptophan and anthranilic acid | HMDB0000197 | C00954 | 899.67 (452.38) | 742.4 (313.93) | 808.38 [606.687,1087.568] | 671.79 [505.087,900.365] | 0.83 | 4.70E-02 | 0.896 |

**Supplementary Table S6. Associations among metabolites class and altered gut bacteria.** P values were corrected for multiple testing using the Benjamini-Hochberg false discovery rate, p<0.05.

| Pairs between metabolites class and bacteria | G0.R | G0.P | G1.R | G1.P |
| --- | --- | --- | --- | --- |
| Amino acids_Unclassified | -0.09127 | 0.519915 | -0.02939 | 0.841131 |
| Cinnamic acids and derivatives_Unclassified | -0.03902 | 0.783611 | 0.169286 | 0.244901 |
| Imidazopyrimidines_Unclassified | -0.01588 | 0.911034 | 0.058469 | 0.689848 |
| Fluorenes_Unclassified | -0.16665 | 0.237677 | 0.156633 | 0.282476 |
| Nucleosides [Fig]_Unclassified | -0.10441 | 0.461333 | -0.09959 | 0.495972 |
| Sulfamic acid derivatives_Unclassified | 0.390079 | 0.004253 | 0.135612 | 0.352847 |
| Lactams_Unclassified | -0.19278 | 0.170929 | -0.01316 | 0.928472 |
| Carboxylic acids and derivatives_Unclassified | 0.078204 | 0.581585 | 0.073367 | 0.616379 |
| Alkaloids derived from tryptophan and anthranilic acid_Unclassified | -0.06309 | 0.656791 | 0.217347 | 0.133575 |
| Amino acids_Alistipes | 0.008538 | 0.9521 | -0.08367 | 0.567594 |
| Cinnamic acids and derivatives_Alistipes | 0.106378 | 0.452902 | 0.198061 | 0.172506 |
| Imidazopyrimidines_Alistipes | 0.073337 | 0.605377 | -0.01061 | 0.942307 |
| Fluorenes_Alistipes | -0.13831 | 0.328165 | 0.057959 | 0.69242 |
| Nucleosides [Fig]_Alistipes | -0.01904 | 0.893431 | -0.2102 | 0.147143 |
| Sulfamic acid derivatives_Alistipes | 0.436524 | 0.001215 | -0.0902 | 0.537639 |
| Lactams_Alistipes | -0.08802 | 0.534921 | 0.034388 | 0.814543 |
| Carboxylic acids and derivatives_Alistipes | 0.142491 | 0.313601 | -0.19296 | 0.184056 |
| Alkaloids derived from tryptophan and anthranilic acid_Alistipes | 0.146675 | 0.299457 | -0.11694 | 0.4236 |
| Amino acids_Butyricimonas | 0.061214 | 0.666399 | 0.012755 | 0.930684 |
| Cinnamic acids and derivatives_Butyricimonas | 0.09562 | 0.500112 | 0.227245 | 0.116352 |
| Imidazopyrimidines_Butyricimonas | 0.097242 | 0.492835 | 0.115 | 0.431379 |
| Fluorenes_Butyricimonas | -0.05302 | 0.708935 | 0.17398 | 0.231868 |
| Nucleosides [Fig]_Butyricimonas | 0.150943 | 0.285456 | -0.13724 | 0.347026 |
| Sulfamic acid derivatives_Butyricimonas | -0.00563 | 0.968376 | 0.32102 | 0.024511 |
| Lactams_Butyricimonas | -0.0006 | 0.996645 | 0.069388 | 0.63568 |
| Carboxylic acids and derivatives_Butyricimonas | 0.204901 | 0.145074 | 0.167245 | 0.250719 |
| Alkaloids derived from tryptophan and anthranilic acid_Butyricimonas | -0.04363 | 0.758771 | 0.024898 | 0.865157 |
| Amino acids_Parabacteroides | 0.216853 | 0.122546 | -0.01112 | 0.939539 |
| Cinnamic acids and derivatives_Parabacteroides | 0.162725 | 0.249073 | -0.02602 | 0.859139 |
| Imidazopyrimidines_Parabacteroides | -0.01904 | 0.893431 | 0.101327 | 0.488459 |
| Fluorenes_Parabacteroides | -0.0508 | 0.720614 | 0.161327 | 0.268118 |
| Nucleosides [Fig]_Parabacteroides | -0.00102 | 0.994249 | -0.1599 | 0.272436 |
| Sulfamic acid derivatives_Parabacteroides | -0.04303 | 0.761978 | 0.148163 | 0.309634 |
| Lactams_Parabacteroides | -0.01733 | 0.90294 | -0.13337 | 0.360947 |
| Carboxylic acids and derivatives_Parabacteroides | 0.177068 | 0.2092 | -0.00918 | 0.950063 |
| Alkaloids derived from tryptophan and anthranilic acid_Parabacteroides | 0.03987 | 0.778994 | 0.209286 | 0.148959 |
| Amino acids_Eubacterium_coprostanoligenes | -0.20336 | 0.148181 | -0.05194 | 0.72302 |
| Cinnamic acids and derivatives_Eubacterium_coprostanoligenes | 0.139418 | 0.32426 | 0.163571 | 0.261427 |
| Imidazopyrimidines_Eubacterium_coprostanoligenes | -0.01776 | 0.900562 | 0.061633 | 0.67398 |
| Fluorenes_Eubacterium_coprostanoligenes | 0.106719 | 0.451445 | 0.052347 | 0.720931 |
| Nucleosides [Fig]_Eubacterium_coprostanoligenes | -0.10057 | 0.478072 | 0.031531 | 0.829713 |
| Sulfamic acid derivatives_Eubacterium_coprostanoligenes | 0.389396 | 0.004327 | -0.00582 | 0.968361 |
| Lactams_Eubacterium_coprostanoligenes | -0.20695 | 0.141009 | -0.00837 | 0.954497 |
| Carboxylic acids and derivatives_Eubacterium_coprostanoligenes | -0.08495 | 0.54933 | 0.096633 | 0.508924 |
| Alkaloids derived from tryptophan and anthranilic acid_Eubacterium_coprostanoligenes | -0.18603 | 0.186685 | 0.065204 | 0.656233 |
| Amino acids_Ruminococcus | -0.066 | 0.642049 | -0.07765 | 0.595876 |
| Cinnamic acids and derivatives_Ruminococcus | 0.129685 | 0.359501 | 0.07102 | 0.627732 |
| Imidazopyrimidines_Ruminococcus | 0.048237 | 0.73417 | 0.1 | 0.494199 |
| Fluorenes_Ruminococcus | -0.12303 | 0.384906 | -0.02388 | 0.870636 |
| Nucleosides [Fig]_Ruminococcus | -0.14787 | 0.295492 | -0.21867 | 0.131162 |
| Sulfamic acid derivatives_Ruminococcus | 0.396995 | 0.003569 | 0.180306 | 0.215068 |
| Lactams_Ruminococcus | -0.20891 | 0.137192 | -0.03092 | 0.832972 |
| Carboxylic acids and derivatives_Ruminococcus | -0.03552 | 0.802616 | 0.02602 | 0.859139 |
| Alkaloids derived from tryptophan and anthranilic acid_Ruminococcus | -0.08597 | 0.544507 | 0.169694 | 0.243748 |
| Amino acids_Subdoligranulum | -0.15274 | 0.279706 | 0.023265 | 0.873925 |
| Cinnamic acids and derivatives_Subdoligranulum | -0.12968 | 0.359501 | -0.00582 | 0.968361 |
| Imidazopyrimidines_Subdoligranulum | 0.08751 | 0.53731 | 0.157959 | 0.278368 |
| Fluorenes_Subdoligranulum | -0.0817 | 0.564738 | 0.012959 | 0.929578 |
| Nucleosides [Fig]_Subdoligranulum | -0.0029 | 0.983706 | -0.20592 | 0.155759 |
| Sulfamic acid derivatives_Subdoligranulum | 0.291215 | 0.03621 | -0.00316 | 0.98279 |
| Lactams_Subdoligranulum | -0.035 | 0.805407 | -0.03867 | 0.791915 |
| Carboxylic acids and derivatives_Subdoligranulum | -0.1255 | 0.375339 | -0.04592 | 0.754056 |
| Alkaloids derived from tryptophan and anthranilic acid_Subdoligranulum | 0.014685 | 0.917707 | 0.055816 | 0.703259 |
| Amino acids_Roseburia | -0.12465 | 0.378622 | 0.159388 | 0.273989 |
| Cinnamic acids and derivatives_Roseburia | -0.11842 | 0.403101 | 0.098776 | 0.499528 |
| Imidazopyrimidines_Roseburia | 0.076838 | 0.58822 | 0.292857 | 0.041142 |
| Fluorenes_Roseburia | 0.093059 | 0.511714 | -0.06724 | 0.646175 |
| Nucleosides [Fig]_Roseburia | -0.14753 | 0.296622 | 0.22051 | 0.127875 |
| Sulfamic acid derivatives_Roseburia | 0.116793 | 0.40962 | 0.049184 | 0.737172 |
| Lactams_Roseburia | -0.0788 | 0.578692 | 0.124694 | 0.393292 |
| Carboxylic acids and derivatives_Roseburia | -0.11073 | 0.434512 | -0.08643 | 0.554863 |
| Alkaloids derived from tryptophan and anthranilic acid_Roseburia | 0.092803 | 0.512881 | 0.23051 | 0.111057 |
| Amino acids_Coprococcus | -0.33459 | 0.015333 | 0.057653 | 0.693964 |
| Cinnamic acids and derivatives_Coprococcus | -0.14915 | 0.291283 | 0.216633 | 0.134888 |
| Imidazopyrimidines_Coprococcus | -0.07112 | 0.616368 | 0.076327 | 0.60219 |
| Fluorenes_Coprococcus | -0.08068 | 0.569645 | 0.014082 | 0.923496 |
| Nucleosides [Fig]_Coprococcus | -0.07325 | 0.605798 | 0.075714 | 0.605113 |
| Sulfamic acid derivatives_Coprococcus | 0.293776 | 0.034529 | 0.074184 | 0.61245 |
| Lactams_Coprococcus | -0.19269 | 0.171122 | 0.135714 | 0.352481 |
| Carboxylic acids and derivatives_Coprococcus | -0.14796 | 0.295211 | 0.170714 | 0.240883 |
| Alkaloids derived from tryptophan and anthranilic acid_Coprococcus | -0.22915 | 0.102246 | 0.038367 | 0.793526 |

| Pairs between metabolites and bacteria | G0.R | G0.P | G1.R | G1.P |
| --- | --- | --- | --- | --- |
| 3a,7a-dihydroxycholanoic acid_2_Coprococcus | -0.40912 | 0.0026 | -0.51306 | 0.000164 |
| Chenodeoxycholic acid 3-sulfate_1_Coprococcus | -0.37488 | 0.006177 | -0.49102 | 0.000341 |
| 3a,7a-dihydroxycholanoic acid_3_Coprococcus | -0.18125 | 0.198466 | -0.4498 | 0.001185 |
| Oxalosuccinic acid_Roseburia | -0.03167 | 0.823606 | 0.440102 | 0.001554 |
| Urobilinogen_1_Roseburia | 0.306156 | 0.027289 | 0.425102 | 0.002329 |
| Dl-lactic acid_Ruminococcus | 0.042773 | 0.763353 | -0.41684 | 0.002888 |
| Chenodeoxycholic acid 3-sulfate_1_Unclassified | -0.46888 | 0.000455 | -0.40724 | 0.003682 |
| L-gamma-glutamyl-l-leucine_2_Alistipes | -0.05396 | 0.704013 | -0.4 | 0.004404 |
| Hmba_Subdoligranulum | 0.060958 | 0.667714 | 0.392551 | 0.005273 |
| 3a,7a-dihydroxycholanoic acid_2_Unclassified | -0.50474 | 0.000136 | -0.38 | 0.007077 |
| Hmba_Alistipes | 0.126441 | 0.371748 | 0.372551 | 0.008385 |
| Alpha-hydroxytriazolam_Roseburia | -0.0111 | 0.937755 | 0.368673 | 0.009144 |
| Urobilinogen_1_Alistipes | 0.31512 | 0.02288 | 0.365918 | 0.009719 |
| Asp-leu_3_Alistipes | 0.03065 | 0.829225 | -0.35939 | 0.011209 |
| L-gamma-glutamyl-l-leucine_3_Alistipes | -0.04738 | 0.738706 | -0.35704 | 0.01179 |
| Asp-leu_3_Butyricimonas | -0.16605 | 0.239387 | -0.35643 | 0.011945 |
| 6-thioxanthine 5'-monophosphate_Roseburia | -0.04491 | 0.751912 | 0.355 | 0.012316 |
| Capryloylglycine_Butyricimonas | -0.01426 | 0.920091 | -0.34857 | 0.014107 |
| L-kynurenine_2_Alistipes | -0.15803 | 0.263177 | -0.34735 | 0.014472 |
| Dl-lactic acid_Butyricimonas | -0.07547 | 0.59489 | -0.33908 | 0.017154 |
| Chenodeoxycholic acid 3-sulfate_1_Eubacterium_coprostanoligenes | -0.22906 | 0.102378 | -0.33878 | 0.017261 |
| Guanadrel_Unclassified | -0.02058 | 0.884885 | 0.335816 | 0.018325 |
| L-threonic acid_Alistipes | -0.028 | 0.843778 | 0.33449 | 0.018819 |
| (2r)-3-{[(2-aminoethoxy)(hydroxy)phosphoryl]oxy}-2-hydroxypropyl (9z)-9-hexadecenoate_Unclassified | 0.081192 | 0.567189 | -0.33418 | 0.018935 |
| (2r)-3-{[(2-aminoethoxy)(hydroxy)phosphoryl]oxy}-2-hydroxypropyl (9z)-9-hexadecenoate_Alistipes | 0.157176 | 0.265798 | -0.33316 | 0.019325 |
| 3a,7a-dihydroxycholanoic acid_2_Eubacterium_coprostanoligenes | -0.24759 | 0.076786 | -0.3298 | 0.02066 |
| 3b-hydroxy-5-cholenoic acid_3_Coprococcus | -0.18441 | 0.190625 | -0.32847 | 0.021207 |
| Cyclamic acid_Butyricimonas | -0.00563 | 0.968376 | 0.32102 | 0.024511 |
| Urobilinogen_1_Eubacterium_coprostanoligenes | 0.339623 | 0.01377 | 0.319286 | 0.025339 |
| 3a,7a-dihydroxycholanoic acid_3_Unclassified | -0.23956 | 0.087169 | -0.31867 | 0.025637 |
| Asp-leu_3_Ruminococcus | -0.16153 | 0.252613 | -0.30949 | 0.030469 |
| Isophorone_2_Coprococcus | 0.026637 | 0.851311 | 0.304286 | 0.033528 |
| Methyl (2r,3s)-3-hydroxy-8-methyl-8-azabicyclo[3.2.1]octane-2-carboxylate_Eubacterium_coprostanoligenes | -0.10689 | 0.450717 | -0.30041 | 0.035968 |
| 5-hydroxyindoleacetate_1_Alistipes | -0.11295 | 0.4253 | -0.29959 | 0.036501 |
| Hmba_Parabacteroides | 0.008538 | 0.9521 | 0.297551 | 0.037859 |
| Dl-lactic acid_Roseburia | 0.235892 | 0.092271 | -0.29735 | 0.037997 |
| Hmba_Eubacterium_coprostanoligenes | 0.361564 | 0.008447 | 0.295612 | 0.039188 |
| 3,6,9,12-tetraoxatridecan-1-ol_Subdoligranulum | -0.12636 | 0.372073 | -0.29367 | 0.040555 |
| (2r)-3-{[(2-aminoethoxy)(hydroxy)phosphoryl]oxy}-2-hydroxypropyl (9z)-9-hexadecenoate_Subdoligranulum | 0.044651 | 0.753282 | -0.29245 | 0.041439 |
| Eugenitin_Parabacteroides | 0.032528 | 0.81893 | 0.290816 | 0.042641 |
| 3b-hydroxy-5-cholenoic acid_2_Coprococcus | -0.40937 | 0.002582 | -0.28816 | 0.044656 |
| Niclosamide_Parabacteroides | 0.173995 | 0.217341 | 0.287959 | 0.044814 |
| Guaiacol sulfate_1_Coprococcus | -0.17852 | 0.205431 | 0.286531 | 0.045933 |
| Uridine_2_Roseburia | -0.11935 | 0.399355 | 0.285816 | 0.046502 |
| 4-vinylguaiacol sulfate_Alistipes | -0.10057 | 0.478072 | -0.28327 | 0.048579 |
| Uric acid_1_Roseburia | 0.055152 | 0.697766 | 0.282143 | 0.049516 |

**Supplementary Table S7. Associations among altered metabolites and gut bacteria.** P values were corrected for multiple testing using the Benjamini-Hochberg false discovery rate, p<0.05, NA, not significant.

| microbes | metabolite | spearman_correlation | p.value | sig |
| --- | --- | --- | --- | --- |
| Haemophilus | Oxalosuccinic.acid | 0.208005 | 0.037832 | * |
| Alistipes | Oxalosuccinic.acid | -0.2275 | 0.022826 | * |
| Phascolarctobacterium | Oxalosuccinic.acid | -0.21506 | 0.031657 | * |
| Bacteroides | Pipecolic.acid | -0.24646 | 0.013441 | * |
| Alistipes | Indole.3.acetic.acid | -0.20278 | 0.04303 | * |
| Alistipes | L.kynurenine | -0.28675 | 0.003823 | ** |
| Sutterella | Indole.3.acetic.acid | 0.266172 | 0.007435 | ** |
| Ruminococcus | Pipecolic.acid | 0.344017 | 0.000457 | *** |
| Alistipes | X5.hydroxyindoleacetate | -0.34928 | 0.000368 | *** |
| Butyricimonas | Corticosterone | -0.11561 | 0.252034 | NA |
| Oscillibacter | Corticosterone | 0.08414 | 0.405239 | NA |
| Fusicatenibacter | Corticosterone | 0.002064 | 0.983739 | NA |
| Haemophilus | Corticosterone | -0.10842 | 0.282918 | NA |
| Sutterella | Corticosterone | 0.092776 | 0.358576 | NA |
| Parasutterella | Corticosterone | -0.12656 | 0.209577 | NA |
| Lachnospiracea_incertae_sedis | Corticosterone | -0.02558 | 0.800538 | NA |
| Ruminococcus | Corticosterone | -0.01778 | 0.860588 | NA |
| Megamonas | Corticosterone | -0.00521 | 0.959005 | NA |
| Alistipes | Corticosterone | -0.03718 | 0.713404 | NA |
| Fusobacterium | Corticosterone | -0.03434 | 0.734454 | NA |
| Blautia | Corticosterone | -0.03403 | 0.73681 | NA |
| Escherichia | Corticosterone | -0.0137 | 0.892366 | NA |
| Phascolarctobacterium | Corticosterone | 0.131434 | 0.19241 | NA |
| Parabacteroides | Corticosterone | -0.05534 | 0.584445 | NA |
| Clostridium_XlVa | Corticosterone | 0.173838 | 0.08368 | NA |
| Roseburia | Corticosterone | 0.00976 | 0.923226 | NA |
| Faecalibacterium | Corticosterone | -0.10906 | 0.280084 | NA |
| Prevotella | Corticosterone | 0.080156 | 0.427919 | NA |
| Bacteroides | Corticosterone | -0.04383 | 0.665013 | NA |
| Others | Corticosterone | -0.06579 | 0.515454 | NA |
| Butyricimonas | Oxalosuccinic.acid | 0.014828 | 0.883589 | NA |
| Oscillibacter | Oxalosuccinic.acid | -0.04782 | 0.636573 | NA |
| Fusicatenibacter | Oxalosuccinic.acid | -0.08763 | 0.385953 | NA |
| Sutterella | Oxalosuccinic.acid | 0.114407 | 0.25704 | NA |
| Parasutterella | Oxalosuccinic.acid | 0.005962 | 0.953056 | NA |
| Lachnospiracea_incertae_sedis | Oxalosuccinic.acid | 0.186515 | 0.063163 | NA |
| Ruminococcus | Oxalosuccinic.acid | -0.05826 | 0.564786 | NA |
| Megamonas | Oxalosuccinic.acid | 0.12463 | 0.216663 | NA |
| Fusobacterium | Oxalosuccinic.acid | -0.01067 | 0.916119 | NA |
| Blautia | Oxalosuccinic.acid | 0.018996 | 0.851198 | NA |
| Escherichia | Oxalosuccinic.acid | -0.15865 | 0.114887 | NA |
| Parabacteroides | Oxalosuccinic.acid | -0.0856 | 0.397106 | NA |
| Clostridium_XlVa | Oxalosuccinic.acid | 0.078481 | 0.437667 | NA |
| Roseburia | Oxalosuccinic.acid | 0.059163 | 0.558745 | NA |
| Faecalibacterium | Oxalosuccinic.acid | -0.02188 | 0.828898 | NA |
| Prevotella | Oxalosuccinic.acid | 0.149224 | 0.138398 | NA |
| Bacteroides | Oxalosuccinic.acid | -0.12446 | 0.217293 | NA |
| Others | Oxalosuccinic.acid | -0.12553 | 0.213338 | NA |
| Butyricimonas | X3.hydroxyanthranilic.acid | -0.08596 | 0.395146 | NA |
| Oscillibacter | X3.hydroxyanthranilic.acid | -0.07509 | 0.457768 | NA |
| Fusicatenibacter | X3.hydroxyanthranilic.acid | -0.10475 | 0.299643 | NA |
| Haemophilus | X3.hydroxyanthranilic.acid | -0.0071 | 0.944088 | NA |
| Sutterella | X3.hydroxyanthranilic.acid | 0.000928 | 0.992685 | NA |
| Parasutterella | X3.hydroxyanthranilic.acid | -0.15034 | 0.135448 | NA |
| Lachnospiracea_incertae_sedis | X3.hydroxyanthranilic.acid | 0.072728 | 0.472087 | NA |
| Ruminococcus | X3.hydroxyanthranilic.acid | -0.15448 | 0.12488 | NA |
| Megamonas | X3.hydroxyanthranilic.acid | -0.05981 | 0.554473 | NA |
| Alistipes | X3.hydroxyanthranilic.acid | -0.09167 | 0.364348 | NA |
| Fusobacterium | X3.hydroxyanthranilic.acid | 0.080182 | 0.427768 | NA |
| Blautia | X3.hydroxyanthranilic.acid | -0.04261 | 0.673774 | NA |
| Escherichia | X3.hydroxyanthranilic.acid | 0.016345 | 0.871777 | NA |
| Phascolarctobacterium | X3.hydroxyanthranilic.acid | -0.072 | 0.476569 | NA |
| Parabacteroides | X3.hydroxyanthranilic.acid | 0.024456 | 0.809148 | NA |
| Clostridium_XlVa | X3.hydroxyanthranilic.acid | 0.057462 | 0.570123 | NA |
| Roseburia | X3.hydroxyanthranilic.acid | -0.0808 | 0.424218 | NA |
| Faecalibacterium | X3.hydroxyanthranilic.acid | -0.1004 | 0.320261 | NA |
| Prevotella | X3.hydroxyanthranilic.acid | 0.064983 | 0.520655 | NA |
| Bacteroides | X3.hydroxyanthranilic.acid | 0.018851 | 0.852323 | NA |
| Others | X3.hydroxyanthranilic.acid | -0.06736 | 0.505471 | NA |
| Butyricimonas | Pipecolic.acid | 0.013618 | 0.893031 | NA |
| Oscillibacter | Pipecolic.acid | 0.133528 | 0.185359 | NA |
| Fusicatenibacter | Pipecolic.acid | -0.07223 | 0.475118 | NA |
| Haemophilus | Pipecolic.acid | -0.03716 | 0.713608 | NA |
| Sutterella | Pipecolic.acid | 0.049037 | 0.628035 | NA |
| Parasutterella | Pipecolic.acid | -0.02457 | 0.808283 | NA |
| Lachnospiracea_incertae_sedis | Pipecolic.acid | 0.024869 | 0.80599 | NA |
| Megamonas | Pipecolic.acid | -0.0501 | 0.620619 | NA |
| Alistipes | Pipecolic.acid | 0.079121 | 0.433926 | NA |
| Fusobacterium | Pipecolic.acid | -0.09143 | 0.365636 | NA |
| Blautia | Pipecolic.acid | -0.08625 | 0.393534 | NA |
| Escherichia | Pipecolic.acid | -0.04686 | 0.643406 | NA |
| Phascolarctobacterium | Pipecolic.acid | -0.03747 | 0.711258 | NA |
| Parabacteroides | Pipecolic.acid | -0.012 | 0.905673 | NA |
| Clostridium_XlVa | Pipecolic.acid | 0.108925 | 0.280692 | NA |
| Roseburia | Pipecolic.acid | -0.00581 | 0.954265 | NA |
| Faecalibacterium | Pipecolic.acid | 0.001631 | 0.987155 | NA |
| Prevotella | Pipecolic.acid | 0.139363 | 0.166704 | NA |
| Others | Pipecolic.acid | 0.10655 | 0.291375 | NA |
| Butyricimonas | L.phenylalanine | -0.00637 | 0.949843 | NA |
| Oscillibacter | L.phenylalanine | -0.10707 | 0.289027 | NA |
| Fusicatenibacter | L.phenylalanine | -0.09827 | 0.330702 | NA |
| Haemophilus | L.phenylalanine | -0.03209 | 0.75126 | NA |
| Sutterella | L.phenylalanine | 0.005176 | 0.959242 | NA |
| Parasutterella | L.phenylalanine | -0.03995 | 0.693128 | NA |
| Lachnospiracea_incertae_sedis | L.phenylalanine | -0.07256 | 0.473127 | NA |
| Ruminococcus | L.phenylalanine | 0.071888 | 0.477229 | NA |
| Megamonas | L.phenylalanine | -0.04141 | 0.682515 | NA |
| Alistipes | L.phenylalanine | 0.070058 | 0.488548 | NA |
| Fusobacterium | L.phenylalanine | -0.05261 | 0.603189 | NA |
| Blautia | L.phenylalanine | 0.007165 | 0.943597 | NA |
| Escherichia | L.phenylalanine | -0.00803 | 0.936763 | NA |
| Phascolarctobacterium | L.phenylalanine | 0.097006 | 0.336989 | NA |
| Parabacteroides | L.phenylalanine | 0.120199 | 0.233574 | NA |
| Clostridium_XlVa | L.phenylalanine | -0.00654 | 0.948535 | NA |
| Roseburia | L.phenylalanine | 0.053992 | 0.593671 | NA |
| Faecalibacterium | L.phenylalanine | -0.10419 | 0.302242 | NA |
| Prevotella | L.phenylalanine | -0.11791 | 0.242651 | NA |
| Bacteroides | L.phenylalanine | 0.177911 | 0.076578 | NA |
| Others | L.phenylalanine | -0.03123 | 0.757768 | NA |
| Butyricimonas | L.kynurenine | -0.05766 | 0.568789 | NA |
| Oscillibacter | L.kynurenine | -0.15812 | 0.116132 | NA |
| Fusicatenibacter | L.kynurenine | -0.12563 | 0.212967 | NA |
| Haemophilus | L.kynurenine | 0.146576 | 0.145613 | NA |
| Sutterella | L.kynurenine | 0.024377 | 0.809752 | NA |
| Parasutterella | L.kynurenine | 0.065874 | 0.514937 | NA |
| Lachnospiracea_incertae_sedis | L.kynurenine | -0.04245 | 0.674938 | NA |
| Ruminococcus | L.kynurenine | -0.09358 | 0.354405 | NA |
| Megamonas | L.kynurenine | 0.065149 | 0.519584 | NA |
| Fusobacterium | L.kynurenine | 0.175176 | 0.081292 | NA |
| Blautia | L.kynurenine | -0.02421 | 0.811056 | NA |
| Escherichia | L.kynurenine | 0.10271 | 0.309213 | NA |
| Phascolarctobacterium | L.kynurenine | -0.12198 | 0.226664 | NA |
| Parabacteroides | L.kynurenine | -0.10647 | 0.291721 | NA |
| Clostridium_XlVa | L.kynurenine | 0.020257 | 0.841447 | NA |
| Roseburia | L.kynurenine | -0.1906 | 0.057496 | NA |
| Faecalibacterium | L.kynurenine | 0.117066 | 0.246074 | NA |
| Prevotella | L.kynurenine | 0.131772 | 0.19126 | NA |
| Bacteroides | L.kynurenine | -0.08454 | 0.403028 | NA |
| Others | L.kynurenine | -0.18876 | 0.059995 | NA |
| Butyricimonas | X5.hydroxyindoleacetate | -0.06412 | 0.526193 | NA |
| Oscillibacter | X5.hydroxyindoleacetate | -0.10602 | 0.293815 | NA |
| Fusicatenibacter | X5.hydroxyindoleacetate | -0.13793 | 0.171161 | NA |
| Haemophilus | X5.hydroxyindoleacetate | 0.03104 | 0.759169 | NA |
| Sutterella | X5.hydroxyindoleacetate | 0.039358 | 0.697437 | NA |
| Parasutterella | X5.hydroxyindoleacetate | 0.059573 | 0.556019 | NA |
| Lachnospiracea_incertae_sedis | X5.hydroxyindoleacetate | -0.04108 | 0.68489 | NA |
| Ruminococcus | X5.hydroxyindoleacetate | 0.003301 | 0.973998 | NA |
| Megamonas | X5.hydroxyindoleacetate | 0.057117 | 0.57245 | NA |
| Fusobacterium | X5.hydroxyindoleacetate | 0.162849 | 0.10548 | NA |
| Blautia | X5.hydroxyindoleacetate | 0.026004 | 0.797321 | NA |
| Escherichia | X5.hydroxyindoleacetate | 0.056591 | 0.575999 | NA |
| Phascolarctobacterium | X5.hydroxyindoleacetate | -0.08099 | 0.423084 | NA |
| Parabacteroides | X5.hydroxyindoleacetate | -0.16044 | 0.110804 | NA |
| Clostridium_XlVa | X5.hydroxyindoleacetate | 0.142455 | 0.157403 | NA |
| Roseburia | X5.hydroxyindoleacetate | -0.04353 | 0.667186 | NA |
| Faecalibacterium | X5.hydroxyindoleacetate | 0.026945 | 0.790153 | NA |
| Prevotella | X5.hydroxyindoleacetate | 0.1092 | 0.279469 | NA |
| Bacteroides | X5.hydroxyindoleacetate | -0.09774 | 0.33332 | NA |
| Others | X5.hydroxyindoleacetate | -0.11711 | 0.245893 | NA |
| Butyricimonas | Indole.3.acetic.acid | -0.08719 | 0.388379 | NA |
| Oscillibacter | Indole.3.acetic.acid | 0.01945 | 0.847689 | NA |
| Fusicatenibacter | Indole.3.acetic.acid | -0.02384 | 0.813903 | NA |
| Haemophilus | Indole.3.acetic.acid | 0.131581 | 0.191911 | NA |
| Parasutterella | Indole.3.acetic.acid | -0.12556 | 0.213228 | NA |
| Lachnospiracea_incertae_sedis | Indole.3.acetic.acid | 0.024394 | 0.809625 | NA |
| Ruminococcus | Indole.3.acetic.acid | -0.12746 | 0.206314 | NA |
| Megamonas | Indole.3.acetic.acid | -0.09505 | 0.346892 | NA |
| Fusobacterium | Indole.3.acetic.acid | 0.096219 | 0.340941 | NA |
| Blautia | Indole.3.acetic.acid | 0.19131 | 0.056559 | NA |
| Escherichia | Indole.3.acetic.acid | 0.016597 | 0.869814 | NA |
| Phascolarctobacterium | Indole.3.acetic.acid | 0.073439 | 0.467753 | NA |
| Parabacteroides | Indole.3.acetic.acid | -0.00344 | 0.97292 | NA |
| Clostridium_XlVa | Indole.3.acetic.acid | 0.188774 | 0.059977 | NA |
| Roseburia | Indole.3.acetic.acid | 0.024252 | 0.810712 | NA |
| Faecalibacterium | Indole.3.acetic.acid | 0.13413 | 0.183368 | NA |
| Prevotella | Indole.3.acetic.acid | -0.11851 | 0.240263 | NA |
| Bacteroides | Indole.3.acetic.acid | 0.111387 | 0.269894 | NA |
| Others | Indole.3.acetic.acid | -0.04618 | 0.648251 | NA |
